# *Methylobacterium ajmalii* sp. nov., Isolated From the International Space Station

**DOI:** 10.3389/fmicb.2021.639396

**Published:** 2021-03-15

**Authors:** Swati Bijlani, Nitin K. Singh, V. V. Ramprasad Eedara, Appa Rao Podile, Christopher E. Mason, Clay C. C. Wang, Kasthuri Venkateswaran

**Affiliations:** ^1^Department of Pharmacology and Pharmaceutical Sciences, School of Pharmacy, University of Southern California, Los Angeles, CA, United States; ^2^Jet Propulsion Laboratory, California Institute of Technology, Pasadena, CA, United States; ^3^Department of Plant Science, School of Life Sciences, University of Hyderabad, Hyderabad, India; ^4^WorldQuant Initiative for Quantitative Prediction, Weill Cornell Medicine, New York, NY, United States

**Keywords:** *Methylobacterium*, polyphasic taxomony, ANI, international space station (ISS), whole genome sequencing

## Abstract

Four strains belonging to the family of *Methylobacteriaceae* were isolated from different locations on the International Space Station (ISS) across two consecutive flights. Of these, three were identified as Gram-negative, rod-shaped, catalase-positive, oxidase-positive, motile bacteria, designated as IF7SW-B2^T^, IIF1SW-B5, and IIF4SW-B5, whereas the fourth was identified as *Methylorubrum rhodesianum*. The sequence similarity of these three ISS strains, designated as IF7SW-B2^T^, IIF1SW-B5, and IIF4SW-B5, was <99.4% for 16S rRNA genes and <97.3% for *gyrB* gene, with the closest being *Methylobacterium indicum* SE2.11^T^. Furthermore, the multi-locus sequence analysis placed these three ISS strains in the same clade of *M. indicum.* The average nucleotide identity (ANI) values of these three ISS strains were <93% and digital DNA-DNA hybridization (dDDH) values were <46.4% with any described *Methylobacterium* species. Based on the ANI and dDDH analyses, these three ISS strains were considered as novel species belonging to the genus *Methylobacterium.* The three ISS strains showed 100% ANI similarity and dDDH values with each other, indicating that these three ISS strains, isolated during various flights and from different locations, belong to the same species. These three ISS strains were found to grow optimally at temperatures from 25 to 30°C, pH 6.0 to 8.0, and NaCl 0 to 1%. Phenotypically, these three ISS strains resemble *M. aquaticum* and *M. terrae* since they assimilate similar sugars as sole carbon substrate when compared to other *Methylobacterium* species. Fatty acid analysis showed that the major fatty acid produced by the ISS strains are C_18__:__1_−ω7*c* and C_18__:__1_−ω6*c*. The predominant quinone was ubiquinone 10, and the major polar lipids were diphosphatidylglycerol, phosphatidylcholine, phosphatidylethanolamine, phosphatidylglycerol, and an unidentified lipid. Therefore, based on genomic, phylogenetic, biochemical, and fatty acid analyses, strains IF7SW-B2^T^, IIF1SW-B5, and IIF4SW-B5, are assigned to a novel species within the genus *Methylobacterium*, and the name *Methylobacterium ajmalii* sp. nov. is proposed. The type strain is IF7SW-B2^T^ (NRRL B-65601^T^ and LMG 32165^T^).

## Introduction

The genus *Methylobacterium* contains more species than any other genera within the family *Methylobacteriaceae*, order *Rhizobiales*, and class *Alphaproteobacteria* (Kelly et al., 2014). *Methylobacterium* species are Gram-negative, rod-shaped bacteria. The genus was first proposed by Patt et al. (1976) with *Methylobacterium organophilum* as the type species. The genus *Methylobacterium* was first emended to include facultative methylotrophs that have the ability to grow on methane or methanol as the source of carbon and energy, in addition to sugars and organic acids (Patt et al., 1976). Another taxonomic study classified all other previously known pink-pigmented facultative methylotrophic bacteria under the genus *Methylobacterium* (Green and Bousfield, 1982). Thereafter, 11 species from the genus *Methylobacterium* were redefined into a new genus proposed as *Methylorubrum*, based on 16S rRNA gene sequence, multi-locus sequence analysis (MLSA), genomic, and phenotypic data (Green and Ardley, 2018).

The genus *Methylobacterium* consists of 45 recognized species, which are ubiquitously present in a wide variety of habitats including air, soil, freshwater, and sediments, and can exist either in free-form or associated with plant tissues (Gallego et al.,2005a,b; Kang et al., 2007; Veyisoglu et al., 2013; Kelly et al., 2014; Kwak et al., 2014; Chaudhry et al., 2016; Green and Ardley, 2018; Park et al., 2018). *Methylobacterium* species are involved in nitrogen fixation, phosphate solubilization, abiotic stress tolerance, plant growth promotion, and biocontrol activity against plant pathogens (Madhaiyan et al., 2006; Kumar M. et al., 2016; Parasuraman et al., 2019; Grossi et al., 2020; Krug et al., 2020). For instance, a novel *Methylobacterium* sp. 2A was observed to result in higher density of lateral roots in inoculated potato crops, even under salt stress conditions, compared with control plants that were not inoculated with the bacteria; it was also found to exhibit biocontrol activity against several plant pathogens (Grossi et al., 2020). Furthermore, genomic analysis of *Methylobacterium* sp. 2A revealed the presence of metabolic pathways involved in plant growth promotion, including the genes for producing an auxin, 3-indole acetic acid (Grossi et al., 2020).

In an ongoing Microbial Tracking experiment on the International Space Station (ISS), four strains belonging to the family *Methylobacteriaceae* were isolated (Checinska Sielaff et al., 2019). Some of the *Methylobacterium* species that are phylogenetically related to these ISS strains have been isolated from plant sources (Kang et al., 2007; Chaudhry et al., 2016), indicating that the ISS strains might also display properties related to plant growth promotion. The objectives of this study were to generate whole genome sequences (WGS) and define the phylogenetic novelty of the ISS *Methylobacterium* strains using polyphasic taxonomic analyses. The WGS generated and annotated in this study was used to predict biotechnologically useful genetic determinants.

## Materials and Methods

### Sample Collection and Isolation of Bacteria

Several surface samples (1 m^2^) were collected from the ISS during Microbial Tracking–1 flight experiments from 2015 to 2016. Sample collection, processing, and isolation of cultivable microorganisms were published elsewhere (Checinska Sielaff et al., 2019). Briefly, the polyester wipes used to collect samples and particulates associated with the sampling devices were transported to Earth before being disassociated into sterile phosphate-buffered saline (pH 7.4) solution and plated onto R2A agar medium (Checinska et al., 2015; Checinska Sielaff et al., 2019). The microbial cultures that were grown at 25°C for 7 days were picked from the R2A plates, purified, and stored for further analyses. Distinct colonies (*n* = 4) isolated from three different locations and from a high-efficiency particulate arrestance (HEPA) filter were characterized during this study. These colonies exhibited unique coloration and differential genomic phylogeny. The type strain IF7SW-B2^T^ was isolated during Flight 1 (March 2015) at Location #7, the Overhead-3 panel surface of the Materials Science Research Rack 1, which is used for basic materials research in the microgravity environment of the ISS. The second strain, IIF1SW-B5, was isolated during Flight 2 (May 2015) at Location #1, the Port panel of the Cupola. The Cupola is a small module devoted to the observation of operations outside the ISS, such as robotic activities, spacecraft approaches, and extravehicular activities. The third strain, IIF4SW-B5, was isolated during Flight 2 (May 2015) at Location #4, the surface of the dining table. Even though the main function of the table was for dining, crewmembers also used the table for experimental work. The fourth strain was I1-R3, isolated from the ISS HEPA filter that was returned aboard STS-134/ULF6 in May 2011 and archived as reported earlier (Checinska et al., 2015).

### DNA Extraction and Whole Genome Sequencing Analysis

A biomass of approximately 1 μg wet weight was collected for DNA extraction from each strain after growing on R2A medium at 25°C for 3 days. Total nucleic acid extraction was carried out using ZymoBIOMICS 96 MagBead DNA kit (lysis tubes) (Zymo Research, United States) after bead beating with a Bertin Precellys homogenizer. This was followed by library preparation using the Illumina Nextera Flex Protocol as per Illumina document number 1000000025416 v07. The initial amount of DNA for library preparation was quantified, and 5 to 12 cycles of polymerase chain reaction (PCR) amplification were carried out to normalize the output depending on the input DNA concentration. The amplified genomic DNA fragments were indexed and pooled in 384-plex configuration. Whole-genome shotgun sequencing was performed on a NovaSeq 6000 S4 flowcell PE 2 × 150 platform with a paired-end module. The data were filtered with NGS QC Toolkit v2.3 (Patel and Jain, 2012) for high-quality (HQ) vector- and adaptor-free reads for genome assembly (cutoff read length for HQ, 80%; cutoff quality score, 20). The number of filtered reads obtained were used for assembly with SPAdes 3.14.0 (Bankevich et al., 2012) genome assembler (*k*-mer size- 32 to 72 bases) using default parameters. The genome was annotated using the National Center for Biotechnology Information (NCBI) Prokaryotic Genome Annotation Pipeline 4.11 (Tatusova et al., 2016; Haft et al., 2018). In addition, functional annotation of genome and seed categories were assigned to the genome by implementing the Rapid Annotations using Subsystems Technology (RAST) tool (Aziz et al., 2008).

Genomes of all other strains used in this study were downloaded from NCBI, and the genomic relatedness of ISS strains was identified based on average nucleotide identity (ANI; FastANI) calculations (Jain et al., 2018) and digital DNA-DNA hybridization (dDDH) analysis (Meier-Kolthoff et al., 2013). FastANI was run on all the genomes using the default parameters: Mashmap identity cutoff *I*_0_ = 80%, non-overlapping fragments of size *l* = *3Kb*, and minimum count of reciprocal mappings *τ* = 50.

### Phylogenetic Analysis

Phylogenetic analysis was carried out based on 16S rRNA gene sequencing, and MLSA using six housekeeping genes: ATP synthase F1 beta subunit (*atpD*), DNA strand exchange and recombination gene (*recA*), chaperone gene (*dnaK*), DNA-directed RNA polymerase subunit beta (*rpoB*), glutamine synthetase type I (*glnI*), and DNA gyrase subunit B (*gyrB*), for differentiating *Methylobacterium* species (Green and Ardley, 2018). The 16S rRNA gene sequences of type strains of all 45 *Methylobacterium* species were included in the phylogenetic analysis. In addition, representative species of genus *Methylorubrum*, *Enterovirga*, *Microvirga*, and *Neomegalonema* from family *Methylobacteriaceae*, *Rhizobium* from order *Rhizobiales*, *Caulobacter* from order *Caulobacterales*, in class *Alphaproteobacteria* were included. *Pseudomonas aeruginosa* was selected as the outgroup.

The 16S rRNA gene sequences of all strains were retrieved from NCBI except for the four ISS strains, which were recovered from their respective WGS. Phylogenetic analysis based on housekeeping genes and MLSA was carried out with type strains of 24 *Methylobacterium* species and representative species of other genera. All the gene sequences were retrieved from the genome sequences using RAST v2.0^1^ (Aziz et al., 2008; Overbeek et al., 2014; Brettin et al., 2015). The individual gene sequences for all strains were aligned separately using ClustalW, and then the maximum likelihood tree was generated using MEGA 7.0.26 (Kumar S. et al., 2016). For MLSA, six housekeeping gene sequences for each strain were concatenated manually and aligned using ClustalW, and then the maximum likelihood tree was generated using MEGA 7.0.26 (Kumar S. et al., 2016).

The genome-based tree for the *Methylobacterium* species, including ISS strains and representative species of other genus with available WGS, was constructed using GToTree (Lee, 2019). This tool takes the complete/draft genomes as input and creates a phylogenomic tree based on the prespecified single-copy gene set using a hidden Markov model (HMM); the tool currently has 2,044 unique HMM set as identifiers to cover all three domains of microbial life.

### Phenotypic Characterization of ISS Strains

Phenotypic characterization was performed according to standard protocols (Jones, 1981). Growth of the ISS strains at different temperatures (7, 25, 30, 37, and 45°C) was assessed after incubation on nutrient agar (Sigma, United States) for 7 days. Growth at different pH (4.0–10.0 at intervals of 1.0) was assessed after incubation in nutrient broth (Sigma, United States) at 30°C for 7 days. The pH of the nutrient medium was adjusted using citrate/NaH_2_PO_4_ buffer (pH 4.0–5.0), phosphate buffer (pH 6.0–8.0), and tris buffer (pH 9.0–10.0) (Kim et al., 2019). Salt tolerance was tested by streaking the strains on R2A supplemented with NaCl (0–10% at intervals of 1%) and incubating the plates at 30°C for 7 days. Motility was assessed via the “hanging drop” method by observing the culture under a light microscope (Tindall et al., 2007). Catalase activity was tested by adding 3% hydrogen peroxide to culture grown on R2A at 30°C for 7 days, and effervescence was monitored (Tindall et al., 2007). An oxidase test was carried out in a filter paper soaked with the substrate tetramethyl-p-phenylenediamine dihydrochloride, and coloration was documented (Jurtshuk Jr. and McQuitty, 1976). All other physiological and biochemical tests were carried out using API 20 NE, API 50 CH, and API ZYM kits as per manufacturer’s procedures (bioMérieux, France).

### Chemotaxonomic Analysis

All strains grown in the R2A broth were harvested when growth of the cultures reached around 70% of the maximal optical density (exponential growth phase), and then the cultures were used for analyses of cellular fatty acids, polar lipids, and quinones, which were carried out as described previously (Ramaprasad et al., 2015). Briefly, for cellular fatty acids analysis, 40 mg of bacterial cell pellet from each strain was subjected to a series of four different reagents followed by saponification and methylation of fatty acids, thus enabling their cleavage from lipids. The fatty acid methyl esters (FAME) thus obtained were analyzed by a gas chromatograph equipped with Sherlock MIS software (Microbial ID; MIDI 6.0 version; Agilent: 6850)^2^. The peaks obtained were then labeled, and the equivalent chain length (ECL) values were computed by the Sherlock software.

The polar lipids profile was analyzed by extracting cells with methanol-chloroform-saline (2:1:0.8, v/v/v) from 1 g of freeze-dried bacterial cells. Separation of lipids was performed by two-dimensional chromatography on a silica gel thin-layer chromatography plate (Kieselgel 60 F254; Merck) using chloroform-methanol-water (75:32:4, v/v/v) in the first dimension and chloroform–methanol–acetic acid–water (86:16:15:4, v/v/v/v) in the second dimension. The total polar lipids profile was detected by spraying with 6% ethanolic molybdophosphoric acid. The respiratory isoprenoid quinone was extracted with a chloroform-methanol mixture (2:1, v/v), evaporated under vacuum, re-extracted with acetone, and analyzed using high-performance lipid chromatography as per established methods (Ramaprasad et al., 2018).

## Results and Discussion

This study reports the isolation and identification of four strains belonging to the family *Methylobacteriaceae*, collected from different locations on the ISS. Three of the strains, referred to as IF7SW-B2^T^, IIF1SW-B5, and IIF4SW-B5, were identified based on the traditional and genomic taxonomic approaches. The fourth strain, which was isolated from a HEPA filter and referred to as I1-R3, was identified based on genomic analyses only.

### Phylogenetic Analysis of Novel ISS Strains

**FIGURE 1:**
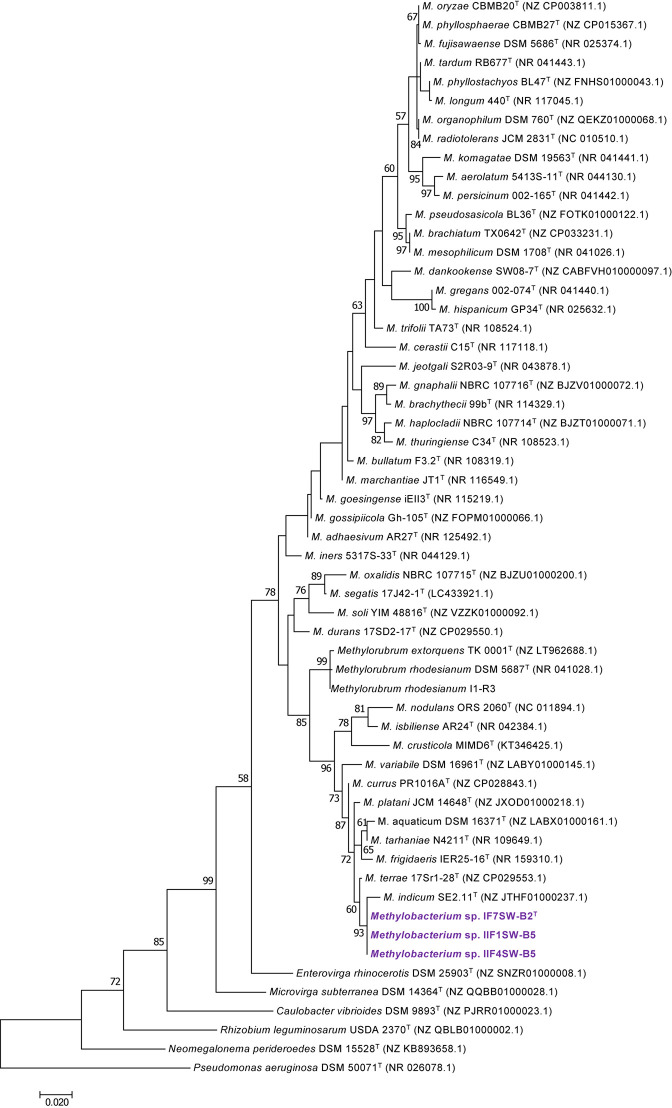
Maximum likelihood phylogenetic tree based on 16S rRNA gene sequences shows the relationship of *Methylobacterium ajmalii* sp. nov. with members of the family *Methylobacteriaceae*. Bootstrap values from 1,000 replications are shown at branch points. Bar, 0.02 substitution per site.

**FIGURE 2:**
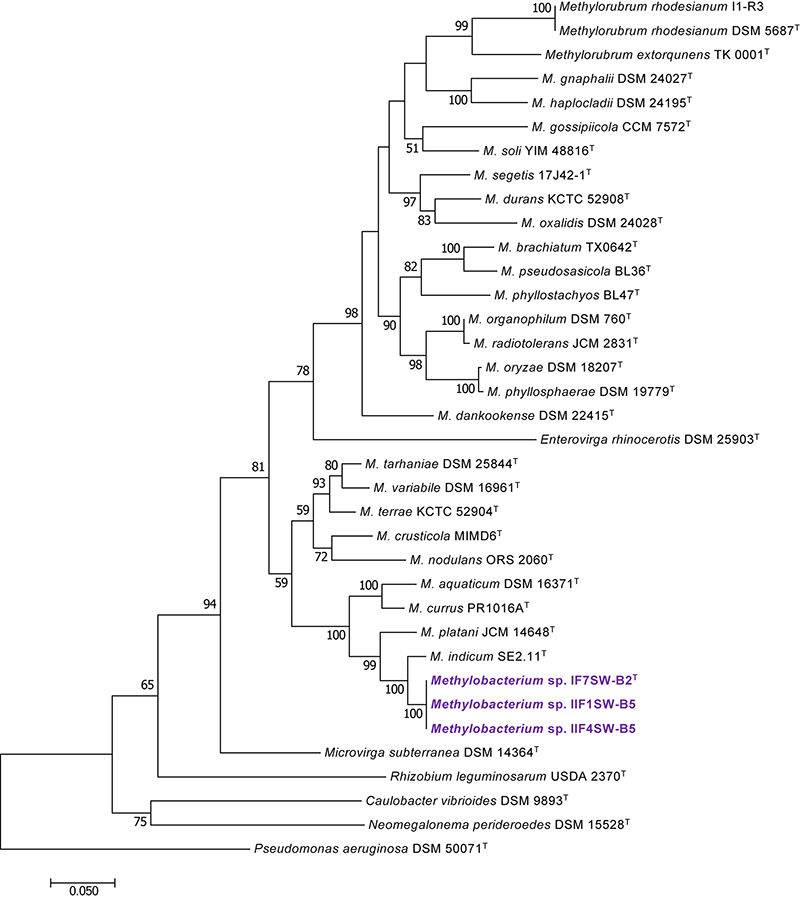
Maximum likelihood phylogenetic tree, based on DNA gyrase gene (*gyrB*) sequences, showing the phylogenetic relationship of *Methylobacterium ajmalii* sp. nov. with members of the family *Methylobacteriaceae*. Bootstrap values from 1,000 replications are shown at branch points. Bar, 0.05 substitution per site.

**FIGURE 3:**
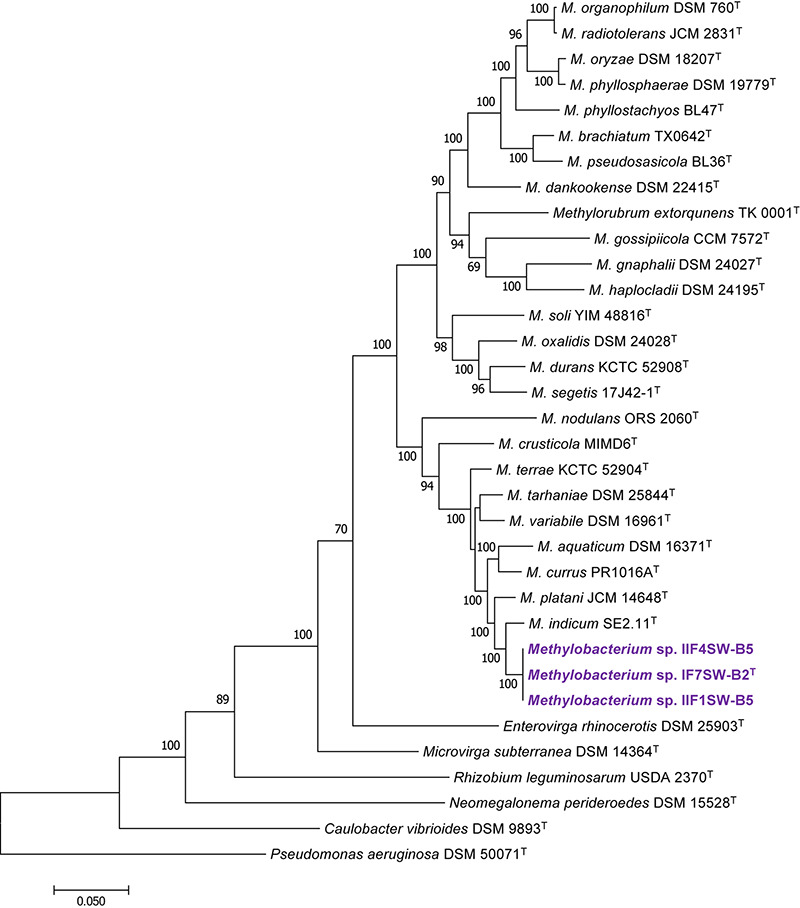
Maximum likelihood phylogenetic tree, based on six gene sequences (*atpD*, *recA*, *dnaK*, *rpoB*, *glnI*, and *gyrB*) concatenated manually, showing the phylogenetic relationship of *Methylobacterium ajmalii* sp. nov. with members of the family *Methylobacteriaceae*. Bootstrap values from 1,000 replications are shown at branch points. Bar, 0.05 substitution per site.

**FIGURE 4:**
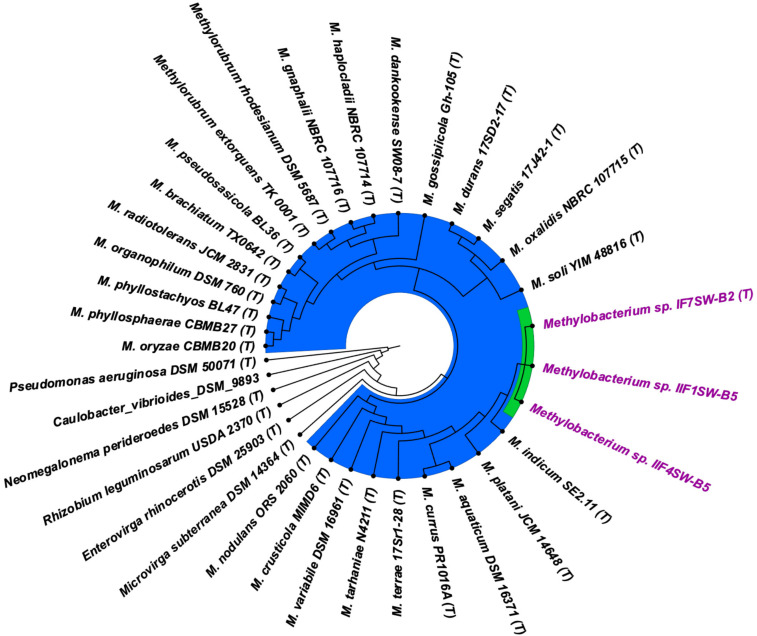
Genome-based phylogenetic tree showing the phylogenetic relationship of *Methylobacterium ajmalii* sp. nov. with members of the family *Methylobacteriaceae*.

To confirm that three of the ISS strains (IF7SW-B2^T^, IIF1SW-B5, and IIF4SW-B5) belong to a novel species, their phylogenetic affiliations were analyzed with other species belonging to the genus *Methylobacterium*. The sequence similarity of these three ISS strains with validly described *Methylobacterium* species was <99.4% for 16S rRNA gene (Supplementary Table 1) and <97.3% for *gyrB* gene with the closest being *M. indicum* SE2.11^T^. Phylogenetic analysis of these three ISS strains was carried out by constructing a maximum likelihood tree based on 16S rRNA (Figure 1), *gyrB* (Figure 2), *atpD* (Supplementary Figure 1), *recA* (Supplementary Figure 2), *dnaK* (Supplementary Figure 3), *rpoB* (Supplementary Figure 4), and *glnI* (Supplementary Figure 5) gene sequences. In addition, MLSA was carried out by concatenating the six housekeeping genes manually (Figure 3). In addition, a phylogenetic tree based on WGS was generated (Figure 4). The phylogenetic trees constructed based on all these genes, MLSA, and WGS showed that these three ISS strains (IF7SW-B2^T^, IIF1SW-B5, and IIF4SW-B5) are clustered together and in the same clade with *M. indicum* SE2.11^T^. The 16S rRNA gene-sequencing, housekeeping gene-based analyses, MLSA, and genome-based tree further supported the concept that these three ISS strains belong to the same species but are closely related to *M. indicum*. In addition, the identity of the ISS strain I1-R3 was further confirmed to be *M. rhodesianum* based on its 16S rRNA gene (Figure 1) and *gyrB* (Figure 2) phylogenetic affiliation to the type strain *M. rhodesianum* DSM 5687^T^.

### Whole Genome Sequence–Based Phylogenetic Analysis

The genomes of the four isolated ISS strains were sequenced, with their draft genome assembled and annotated. The results are summarized in Table 1. The genome varied in size from 6.1 to 6.8 Mbp with GC content between 68 and 71%, similar to other members of the family *Methylobacteriaceae*.

**TABLE 1:** Summary of the draft whole-genome sequences of four strains belonging to the family *Methylobacteriaceae*, isolated from the ISS.

Species/Strain	NCBI Accession no.	Isolation location	No. of scaffolds	Genome size (bp)	N_5_o (bp)	Average Coverage	G + C content (%)	Filtered reads used for assembly (million)	Coding sequences
*Methylobacterium ajmalii IF7SW-B2^T^*	JACWCTOOOOOOOOO	Lab 3 overhead	192	6,802,552	59,313	698	71.07	33.55	6,255
*Methylobacterium ajmalii* IIF1SW-B5	JACWCUOOOOOOOOO	Cupola	193	6,593,618	50,984	754	71.03	36.29	6,076
*Methylobacterium ajmalii* IIF4SW-B5	JACWCVOOOOOOOOO	Dining table	966	6,534,937	10,467	538	70.77	25.01	6,538
*Methylorubrum rhodesianum* I1-R3	JACWCWOOOOOOOOO	HEPA filter	160	6,159,250	98,200	302	68.96	13.07	5,555

Due to higher sequence similarities of three ISS strains with *M. indicum* SE2.11^T^ (99.4% for 16S rRNA gene and 97.3% for *gyr*B gene), the draft genomes of three ISS strains were subjected to ANI and dDDH analysis with other species belonging to family *Methylobacteriaceae* (Table 2). The ANI indices of three ISS strains (IF7SW-B2^T^, IIF1SW-B5, and IIF4SW-B5) with *M. indicum* SE2.11^T^ were 92.7 to 93%, and dDDH values were 45.8 to 46.4%. The ANI and dDDH values obtained for three ISS strains with other *Methylobacterium* species were below the threshold of 95% ANI (Yoon et al., 2017) and 70% dDDH values (Auch et al., 2010), which were established for prokaryotic species delineation. This suggested that these three ISS strains are novel species of the genus *Methylobacterium*. These three ISS strains exhibited ANI and dDDH values around 99–100% with each other, indicating that they belong to the same species. The entire genomes of these three ISS strains, *M. indicum* SE2.11^T^, and *M. platani* PMB02^T^ were aligned to detect their divergence and similarity using the MUMmer 3.0 system (Kurtz et al., 2004). As shown in Supplementary Figure 6, genomes of these three ISS strains aligned perfectly, while the closest genomes of *M. indicum* and *M. platani* exhibited divergence with the ISS type strain IF7SW-B2^T^. Since these three ISS strains were isolated at different time periods and from various locations, their persistence in the ISS environment and ecological significance in the closed systems warrant further study.

**TABLE 2:** Genomic analyses of *Methylobacterium ajmalii* in comparison to other species of the family *Methylobacteriaceae*.

Species (NCBI accession no.)	ANI value (%)			dDDH (%) (Formula 2)		
	IF7SW-B2^T^	IIF1SW-B5	IIF4SW-B5	IF7SW-B2^T^	IIF1SW-B5	IIF4SW-B5
*Methylobacterium ajmalii* IF7SW-B2^T^ (JACWCT000000000)	100	100	100	100	100	99.9
*Methylobacterium ajmalii* IIF1SW-B5 (JACWCUOOOOOOOOO)	100	100	100	100	100	99.9
*Methylobacterium ajmalii* IIF4SW-B5 (JACWCVOOOOOOOOO)	99.7	99.6	100	99.9	99.9	100
*Methylobacterium indicum* SE2.11^T^ (GCA_001043895.1)	93.0	92.9	92.7	45.8	45.8	46.4
*Methylobacterium currus* PR1016A^1^ (GCA_003058325.1)	90.5	90.5	90.8	36.4	36.4	37.5
*Methylobacterium terrae* KCTC 52904^T^ (GCA_003173755.1)	90.4	90.4	90.4	34.8	34.8	35.8
*Methylobacterium platani* JCM 14648^T^^(^GCA_001043885.1)	90.2	90.3	90.4	35.4	35.5	36.4
*Methylobacterium tarhaniae* DSM 25844^T^ (GCA_001043955.1)	89.7	89.5	89.7	34.5	34.6	35.6
Methylobacterium aquaticum DSM 16371^T^ (GCA_001043915.1)	89.0	89.0	89.1	33.6	33.6	34.5
*Methylobacterium variabile* DSM 16961^T^ (GCA_001043975.1)	88.9	88.8	88.9	33.5	33.6	34.4
*Methylobacterium crusticola* MIMD6^T^ (GCA_003574465.1)	84.7	84.9	85.1	26.4	26.4	27.1
*Methylobacterium nodulans* ORS 2060^T^ (GCA_000022085.1)	82.7	82.7	82.8	24.2	24.3	24.9
*Methylobacterium dankookense* DSM 22415^T^ (GCA_902141855.1)	81.6	81.5	81.7	22.9	23.0	23.5
*Methylobacterium segetis* 17J42-1^1^ (GCA_004348265.1)	81.2	81.2	81.4	22.7	22.8	23.4
*Methylobacterium oxalidis* DSM 24028^T^ (GCA_007992195.1)	81.2	81.1	81.2	22.3	22.4	23.0
*Methylobacterium durans* KCTC 52908^T^ (GCA_003173715.1)	81.0	80.9	81.0	22.4	22.5	23.1
*Methylobacterium organophilum* DSM 760^T^ (GCA_003096615.1)	80.9	80.8	81.2	22.4	22.4	22.9
*Methylobacterium radiotolerans* JCM 2831^T^ (GCA_000019725.1)	80.9	80.8	81.2	22.2	22.3	22.8
*Methylobacterium brachiatum* TX0642^T^ (GCA_003697185.1)	80.9	80.9	81.1	22.5	22.5	22.9
*Methylobacterium soli* YIM 48816^T^ (GCA_008806385.1)	80.8	80.7	80.9	22.2	22.2	22.8
*Methylobacterium pseudosasicola* BL36^T^ (GCA_900114535.1)	80.7	80.6	80.8	21.7	21.8	22.3
*Methylorubrum extorqunens* TK 0001^T^ (GCA_900234795.1)	80.6	80.6	80.9	22.0	22.0	22.5
*Methylobacterium oryzae* DSM 18207^T^ (GCA_000757795.1)	80.6	80.4	80.6	21.9	22.0	22.5
*Methylobacterium phyllosphaerae* DSM 19779^T^ (GCA_001936175.1)	80.6	80.5	80.6	21.8	21.9	22.4
Methylobacterium phyllostachyos BL47^T^ (GCA_900103445.1)	80.4	80.4	80.4	21.8	21.8	22.3
Methylobacterium gossipiicola CCM 7572^T^ (GCA_900113485.1)	80.3	80.4	80.4	21.8	21.9	22.5
*Methylobacterium haplocladii* DSM 24195^T^ (GCA_007992175.1)	80.3	80.2	80.4	21.7	21.8	22.3
*Methylobacterium gnaphalii* DSM 24027^T^ (GCA_007992215.1)	79.9	79.8	80.0	21.4	21.5	21.9
*Microvirga subterranea* DSM 14364^T^ (GCA_003350535.1)	79.0	78.9	78.8	20.8	20.8	21.2
*Enterovirga rhinocerotis* DSM 25903^T^ (GCA_004363955.1)	78.1	78.1	78.3	20.8	20.8	20.9

The fourth strain I1-R3 was identified as *M. rhodesianum* based on highly similar 16S rRNA (99.9%), *gyrB* (100%), ANI (98.9%), and dDDH (91.6%) genomic parameters with *M. rhodesianum* DSM 5687^T^. The pigmentation of the strain I1-R3 (light pink) was also different from the novel ISS *Methylobacterium* strains (reddish pink). The ANI and dDDH values between I1-R3 and the three novel ISS *Methylobacterium* strains were ∼82% and 24%, respectively. Hence, genomic and morphological analyses confirmed the phylogenetic affiliation of strain I1-R3 as *M. rhodesianum*. In this communication, phylogenetic affiliations of only IF7SW-B2^T^, IIF1SW-B5, and IIF4SW-B5 strains were presented.

### Phenotypic Characterization of Novel ISS Strains

The minimal information about the ISS strain genome characteristics are given in Supplementary Table 2. The differential phenotypic characteristics of IF7SW-B2^T^, IIF1SW-B5, and IIF4SW-B5 are listed in Table 3, in comparison with other related *Methylobacterium* species. Three strains belonging to *Methylobacterium* sp. nov. are reddish pink–pigmented, Gram-stain-negative, catalase-positive, oxidase-positive, motile, and rod-shaped. These strains grew well on nutrient agar and R2A. These three strains grew optimally at temperatures between 25 and 30°C, were viable only at pH 6.0 to 8.0, and exhibited poor tolerance to salt (0 to 1%). Absence of growth was observed when grown at 7, 37, and 45°C. These strains were positive for assimilation of L-arabinose, D-glucose, maltose, D-mannitol, D-mannose, malic acid, potassium gluconate, and trisodium citrate. These strains also exhibited esterase lipase and trypsin enzymatic activities. The complete results of phenotypic characteristics determined using API 20 NE, API ZYM, and API 50 CH are detailed in Supplementary Tables 3-5, respectively. The majority of the phenotypic characteristics of the ISS strains were similar to other *Methylobacterium* species. Phenotypically, these three ISS strains were different from the closest genomic relative *M. indicum* in assimilating glucose, malic acid, maltose, mannitol, potassium gluconate, and trisodium citrate. Furthermore, unlike *M. indicum*, these ISS strains did not exhibit growth at pH 5.0. In comparison to other *Methylobacterium* species, *M. aquaticum* and *M. terrae* exhibit similar carbon substrate utilization and enzyme production profiles. However, malic acid was assimilated by these ISS strains but not by *M. aquaticum*. Maltose was also utilized by these ISS strains but not by *M. terrae* cells.

**TABLE 3:** Differential phenotypic characteristics of *Methylobacterium ajmalii* and related species of genus *Methylobacterium*.

Characteristic	1	2	3	4	5	6	7	8	9
Growth temperature (°C)	25-30*	18-42	20-30	20-30	20-30	10-37	18-37	20-37	10-40
Growth pH	6.0-8.0	5.0-9.0	5.0-7.0	5.0-8.0	6.0-8.0	4.0-9.0	6.0-8.0	5.0-8.0	4.0-7.0
Catalase	+	W	+	W	+	+	+	+	+
Oxidase	+	+	−	−	+	+	+	−	+
Reduction of nitrate to nitrite	−	w	w	w	w	−	−	+	W
Starch hydrolysis	−	−	+	−	−	+	+	−	−
Assimilation (API-20NE) of:									
D-glucose	+	w	W	+	−	+	+	−	−
L-arabinose	+	+	+	−	+	+	+	+	+
D-mannose	+	−	W	−	−	+	+	−	−
D-mannitol	+	−	+	−	−	-	+	−	−
Maltose	+	−	+	−	−	−	−	−	−
Potassium gluconate	+	+	+	−	+	−	−	−	−+
Malic acid	+	+	−	+	−	+	+	−	w
Trisodium citrate	+	+	−	−	−	+	+	−	w
Phenyl acetic acid	−	.-	W	−	−	−	+	−	−
Enzymatic activity (API-ZYM) of:									
Esterase lipase	+	+	W	w	+	−	+	N.D.	+
Cystine arylamidase	−	+	W	w	w	+	+	N.D.	−
Trypsin	+	+	W	w	w	−	+	N.D.	−

Strains: 1: *Methylobacterium* sp. nov. (*n* = 3, where n: IF7SW-B2^*T*^, IIF1SW-B5 and IIF4SW-B5) (this study); 2: *M. currus* PR1016A^*T*^ (Park et al., 2018); 3: *M. aquaticum* DSM 16371^*T*^ (Gallego et al., 2005a; Park et al., 2018); 4: M. variable DSM 16961^*T*^ (Gallego et al., 2005b; Park et al., 2018); 5: M. platani JCM 14648^*T*^ (Kang et al., 2007; Park et al., 2018); 6: M. tarhaniae DSM 25844^*T*^ (Veyisoglu et al., 2013; Kim et al., 2019); 7: M. terrae KCTC 52904^*T*^ (Kim et al., 2019); 8: M. indicum SE2.11^*T*^ (Chaudhry et al., 2016); 9: M. frigidaeris IER25-16^*T*^ (Lee and Jeon, 2018). w, weakly positive; +, Positive; -, Negative; N.D., not determined. All strains exhibited 0-1% NaCl tolerance and were motile. *Growth was tested at 7, 25, 30, 37, and 45°C and cells were grown optimally at 25 and 30°C but no growth was observed at 7, 37, and 45°C. Growth lower than 25°C might be possible but not tested.

The main phenotypic characteristics of the ISS strains IF7SW-B2^T^, IIF1SW-B5, and IIF4SW-B5 were in accordance with the description of the genus *Methylobacterium*, with the most important being reddish pink pigmentation (Green and Bousfield, 1982). The optimum growth conditions (temperature, pH, salt tolerance) of the ISS *Methylobacterium* strains were similar to other members belonging to the genus *Methylobacterium*. Also, these three ISS strains shared the properties of exhibiting catalase activity and motility with other *Methylobacterium* species. However, the three novel ISS *Methylobacterium* strains differed from other members of the genus *Methylobacterium* in some of the phenotypic characteristics, as shown in Table 3. For instance, they exhibited properties like assimilation of certain sugars, which was absent in some of the *Methylobacterium* species. They also did not show cystine arylamidase activity as opposed to several related *Methylobacterium* species.

### Chemotaxonomic Characterization of Novel ISS Strains

The FAME profiling of three ISS strains and other related *Methylobacterium* species are given in Table 4. The major fatty acids in these ISS strains were C_18__:__1_ ω7*c* and/or C_18__:__1_ ω6*c* (Sum in Feature 8; 82 to 85%) with small amounts of C_18__:__0_ 3-OH, C_16__:__0_, C_17__:__0_, Sum in Feature 3, Sum in Feature 2, C_18__:__0_ and C_12__:__0_, and traces of C_11__:__0_, C_13__:__0_, and C_14__:__0__._ The fatty acids, C_18__:__1_ ω7*c* and/or C_18__:__1_ ω6*c*, were observed to be dominant in these ISS strains, similar to other species. However, complete FAME profiles were not consistent among *Methylobacterium* species and some significant differences in the proportions of certain fatty acids were observed (Table 4). The notable difference in the FAME profile was the lower abundance of C_18__:__1_ ω7*c* in *M. indicum* (46%) when compared with these ISS strains (82 to 85%).

**TABLE 4:** Percentage of total cellular fatty acids from *Methylobacterium ajmalii* and related species of genus *Methylobacterium*.

Fatty acids	1	2	3	4	5	6	7	8	9	10	11
C9:0	-	-	-	0.2	-	0.5	-	-	-	-	-
C11:0	0.31	0.66	0.32	0.7	-	0.8	-	-	-	-	-
C12:0	1.06	0.75	1.81	-	-	-	tr	-	-	1.29	7.0
C13:0	0.76	0.65	0.45	-	-	-	-	-	-	-	-
C14:0	0.71	0.42	0.49	0.28	tr	0.9	tr	-	tr	1.18	-
C16:0	3.02	2.66	2.22	8.7	4.1	8.0	7.6	5.7	5.8	6.16	4.2
C17:0	3.09	2.32	2.02	0.4	-	-	-	-	-	1.07	-
C18:0	1.09	0.71	0.57	5.9	1.0	6.6	1.71	2.8	1.6	2.43	2.8
C19:0	-	-	-	-	tr	-	-	-	tr	-	-
C_8__:_o3-OH	-	-	-	1.1	-	1.5	-	-	-	-	-
iso- C_10__:__0_	-	-	-	-	2.1	-	-	-	2.4	-	-
C10:02-OH	-	-	-	0.2	-	0.4	-	-	-	-	-
C11:03-OH	-	-	-	-	tr	-	-	-	tr	-	-
C_12__:__1_ at 11-12	-	-	-	-	tr	-	-	-	tr	-	-
iso- C_13__:__0_	-	-	-	-	tr	-	-	-	tr	2.46	-
C13:0 2-OH	-	-	-	-	1.3	-	-	-	-	-	-
anteiso- C14:0	-	-	-	-	-	-	-	-	-	1.94	-
C14:1-ω5c	-	-	-	-	2.4	-	tr	-	2.4	2.21	-
anteiso- Ci_5__:_o	-	-	-	-	tr	-	-	-	1.2	3.10	-
iso- C_15__:__0_	-	-	-	-	-	-	-	-	-	2.22	-
iso- C_15__:__0_ 3-OH	-	-	-	-	-	-	-	-	-	-	1.6
iso- C_15__:__1_ F	-	-	-	-	tr	-	-	-	tr	-	-
iso- C_15__:__1_ G	-	-	-	-	-	-	-	-	-	1.86	-
C16:0 N-alcohol	-	-	-	-	-	-	tr	-	-	-	-
C16:1- ω5c	-	-	-	-	4.9	-	-	-	4.3	-	-
anteiso- C17:0	-	-	-	-	-	-	-	-	-	1.80	-
anteiso- C17:1-ω9c	-	-	-	-	tr	-	-	-	Tr	2.07	-
iso- C17:1-ω5c	-	-	-	-	-	-	-	-	-	2.86	-
iso- C_17__:__0_ 3-OH	-	-	-	-	tr	-	-	-	tr	1.76	-
C17:1- ω7c	-	-	-	-	tr	-	-	-	tr	-	-
iso-C18:0	0.58	0.46	0.40	-	-	-	-	-	-	-	-
iso-C18:1 H	-	-	-	-	tr	-	-	-	tr	-	-
C18:0- ω5c	0.26	0.28	0.26	-	-	0.7	-	-	-	-	-
C18:1- ω5c	-	-	-	0.6	-	-	-	-	-	-	-
C18:1- ω9c	-	-	-	-	-	-	tr	-	-	-	-
C18:0-3OH	3.14	2.41	3.84	2.4	1.4	1.7	3.46	-	2.0	-	4.9
C18:3- ω6c	-	-	-	-	-	-	tr	-	-	4.99	-
iso-C19:0	-	-	-	-	tr	-	-	-	-	-	-
C19:0 10-methyl	-	-	-	-	-	-	tr	-	-	-	-
C19:0 cyclo- ω8c	-	-	-	-	-	-	-	-	-	-	1.5
C20:1- ω7c	-	-	-	0.4	-	-	-	-	-	-	-
C20:2- ω6,9c	-	-	-	-	-	-	-	-	-	-	1.5
Sum In Feature 8*	82.09	85.08	83.97	66.7	57.6	60.3	81.0	86.4	57.5	46.03	73.8
Sum In Feature 5*	-	-	-	0.6	-	0.7	-	-	-	-	-
Sum In Feature 3*	2.43	2.05	2.37	1.3	10.3	1.0	2.24	1.9	11.2	-	0.9
Sum In Feature 2*	2.17	1.99	1.78	1.8	2.2	2.1	1.98	2.8	1.5	3.22	1.9

Strains: 1: IF7SW-B2^*T*^ (this study), 2: IIF1SW-B5 (this study); 3: IIF4SW-B5 (this study); 4: *M. currus* PR1016A^*T*^ (Park et al., 2018); 5: *M. aquaticum* DSM 16371^*T*^ (Kim et al., 2019); 6*: M. variable* DSM 16961^*T*^ (Park et al., 2018); 7: *M. platani* JCM 14648^*T*^ (Kang et al., 2007); 8: *M. tarhaniae* DSM 25844^*T*^ (Kim et al., 2019); 9: *M. terrae* KCTC 52904^*T*^ (Kim et al., 2019); 10: *M. indicum* SE2.11^*T*^ (Chaudhry et al., 2016); 11: *M. frigidaeris* IER25-16^*T*^ (Lee and Jeon, 2018). -: Not detected, tr: trace amount (<1%). *Summed features represent groups of two or three fatty acids that cannot be separated using the MIDI system. Summed feature 2 (iso-C16:1 I and/or C14:0 3-OH), summed feature 3 (C16:1 ω7*c* and/or C16:1 ω6*c*), Sum In Feature 5 comprises of C18:0-ω6,9c and ante-C18:0 and summed feature 8 (C18:1 ω7*c* and/or C18:1ω6*c*).

These ISS strains contained Q-10 as the major respiratory isoprenoid quinone, which is common in members of the genus *Methylobacterium*. The polar lipids present in these three strains were diphosphatidylglycerol (DPG), phosphatidylglycerol (PG), phosphatidyl choline (PC), phosphatidyl-ethanolamine (PE), and an unidentified lipid (Supplementary Figure 7). The total polar lipid profile of these ISS strains was consistent with their close relatives, predominated with phospholipids, DPG, PG, and PE. Furthermore, the chemotaxonomic data together with the results of the genomic and phylogenetic analysis support the affiliation of strains IF7SW-B2^T^, IIF1SW-B5, and IIF4SW-B5 to the genus *Methylobacterium*.

### Functional Characteristics of the Novel ISS Strain

The genome of the ISS strain IF7SW-B2^T^, type strain, was annotated and analyzed to determine biotechnologically important genetic determinants. The whole genome and annotation analysis predicted a total of 6,531 genes in the assembled draft genome. Among these, 1,430 fell into various RAST categories, contributing to 2,067 predicted features described in Table 5. All the 1,430 feature and subsystems have been documented in Supplementary Data 1. A major fraction of the annotated genes was composed of amino acids and derivatives (408), carbohydrate metabolism (246), protein metabolism (198), genes associated with cofactors, vitamins, prosthetic groups, pigments metabolism (190), and respiration (151) (Table 5). Genes responsible for motility and chemotaxis (95), metabolism of aromatic compounds (47), and stress response (72) were also observed.

**TABLE 5:** Genes belonging to different functional categories based on annotation generated using RAST for *Methylobacterium ajmalii* IF7SW-B2^T^.

Functional description	Predicted genes*
Cofactors, Vitamins, Prosthetic Groups, Pigments	190
Cell Wall and Capsule	26
Virulence, Disease, and Defense	61
Potassium metabolism	10
Photosynthesis	11
Miscellaneous	17
Phages, Prophages, Transposable elements, Plasmids	25
Membrane Transport	69
RNA Metabolism	40
Nucleosides and Nucleotides	92
Protein Metabolism	198
Cell Division and Cell Cycle	2
Motility and Chemotaxis	95
Regulation and Cell signaling	49
Secondary Metabolism	5
DNA Metabolism	99
Fatty Acids, Lipids, and Isoprenoids	94
Nitrogen Metabolism	14
Dormancy and Sporulation	1
Respiration	151
Stress Response	72
Metabolism of Aromatic Compounds	47
Amino Acids and Derivatives	408
Sulfur Metabolism	17
Phosphorus Metabolism	28
Carbohydrates	246

*Total protein coding genes as per annotated genome.

Based on the genome annotation, genes for nitrogen metabolism were predicted in the genome of the ISS strain IF7SW-B2^T^. Most of the subsystem features aligned with the ammonia assimilation pathway (11 genes), which is a preferred nitrogen source for the bacteria (Leigh and Dodsworth, 2007). In addition, metabolic factors similar to high-affinity phosphate transporter and control of Pho regulon were also identified in the ISS strain IF7SW-B2^T^ (Wanner, 1993, 1996). Interestingly, a higher number of stress tolerance genes, especially the oxidative stress response factors, were observed in the ISS strain IF7SW-B2^T^ when compared with other novel species isolated from the ISS; *Methylobacterium* sp. IF7SW-B2^T^ exhibited 58 features, whereas 36 features were identified in *Solibacillus kalamii* (Seuylemezian et al., 2017) and 18 features were identified in *Kalamiella piersonii* (Singh et al., 2019). The results obtained agree with the previous reports that showed altered regulation of the stress response factors in microorganisms, in the presence of microgravity conditions (Orsini et al., 2017; Aunins et al., 2018). Further studies on the role of oxidative stress in species selection are warranted. The WGS assembly of these three ISS strains reported here will enable the comparative genomic characterization of ISS isolates with Earth counterparts in future studies. This will further aid in the identification of genetic determinants that might potentially be responsible for promoting plant growth under microgravity conditions and contribute to the development of self-sustainable plant crops for long-term space missions in future.

### Genes Essential for Interaction With Plants in the ISS Strain

A thorough genomic analysis of the ISS strain IF7SW-B2^T^ revealed the presence of genes that have been involved in promoting plant growth. The isopentenyl tRNA transferase (*miaA*) essential for cytokinin production reported in *M. aquaticum* strain 22A (Tani et al., 2015) was also found in genome of the ISS strain IF7SW-B2^T^ with high similarity. The product of the *miaA* gene was reported to be responsible for isopentenylation of a specific adenine in some tRNAs and confirmed the secretion of zeatin originated from tRNA in *M. extorquens* (Koenig et al., 2002). Furthermore, multiple components of the cobalamin synthesis pathway, such as cobalamin biosynthesis protein BluB, L-threonine 3-O-phosphate decarboxylase (EC 4.1.1.81), adenosylcobinamide-phosphate guanylyltransferase (EC 2.7.7.62), cobyric acid synthase (EC 6.3.5.10), nicotinate-nucleotide—dimethylbenzimidazole phosphoribosyltransferase (EC 2.4.2.21), adenosylcobinamide-phosphate synthase (EC 6.3.1.10), cob(I)alamin adenosyltransferase. (EC 2.5.1.17), cobalamin synthase (EC 2.7.8.26), and adenosylcobinamide kinase (EC 2.7.1.156), were identified in genome of the ISS strain IF7SW-B2^T^. The metabolic pathway for cobalamin synthesis predicted in the ISS strain is presented (Supplementary Figure 8). Supporting this prediction, previous study also reported that *Methylobacterium* strains harbor genes involved in the production of a variety of vitamins, such as cobalamin, biotin, thiamin, and riboflavin, indicating the potential of methylobacteria promoting algal growth (Krug et al., 2020). In addition, genes associated with siderophore production, i.e., ferric siderophore transport system, biopolymer transport protein ExbB, and multiple flagellar proteins, were identified in genome of the ISS strain IF7SW-B2^T^ and are listed in the Supplementary Figure 8. Genes involved in iron acquisition and metabolism in which microalgae benefit from bacterial siderophores have been reported previously in *Methylobacterium* spp. (Krug et al., 2020). In the “carbon for iron mutualism” concept, algae assimilated iron complexed in bacterial siderophores and in return provided essential dissolved organic matter for the bacteria (Amin et al., 2015). Similar studies are warranted to confirm the plant-growth promoting activities in the IF7SW-B2^T^ ISS strain.

In summary, the phylogenetic and genetic distinctiveness and differential phenotypic properties were sufficient to categorize these three ISS strains as members of a species distinct from other recognized *Methylobacterium* species. Therefore, on the basis of the data presented, strains IF7SW-B2^T^, IIF1SW-B5, and IIF4SW-B5 represent a novel species of the genus *Methylobacterium*, for which the name *Methylobacterium ajmalii* sp. nov. is proposed. The type strain is IF7SW-B2^T^ (NRRL B-65601^T^ and LMG 32165^T^).

### Description of *Methylobacterium ajmalii* sp. nov.

*Methylobacterium ajmalii* (aj.ma’li.i. N.L. gen. n. *ajmalii* named after Ajmal Khan, a renowned Indian scientist on biodiversity). Cells are Gram-stain-negative, aerobic, and motile rods showing oxidase- and catalase-positive reactions. Cells are 1.6–1.8 μm wide and 2.2–3.2 μm long. Colonies on R2A agar are reddish pink–pigmented, circular, convex, and smooth, with a diameter of approximately 0.6–1.0 mm after 3 days of incubation on R2A agar. Growth occurs at 25–30°C (optimum, 30°C), at pH 6.0–8.0 (optimum, pH 7.0) and in the presence of 0–1.0% (w/v) NaCl (optimum, 0%). In API ZYM tests, the strain is positive for Alkaline phosphatase, Esterase (C4), Esterase lipase (C8), Leucine arylamidase, Trypsin, Acid phosphatase, and Naphthol-AS-BI-phosphohydrolase, but negative for other enzyme activities. Cells utilize Adipic acid, D-glucose, D-maltose, D-mannitol, D-mannose, L-arabinose, Malic acid, N-acetyl-glucosamine, Potassium gluconate, and Trisodium citrate for growth, but not other substrates in API 20NE. Cells are capable of weakly fermenting inulin and D-melezitose as observed in API 50 CH. Ubiquinone Q-10 is the predominant respiratory isoprenoid quinone. The major fatty acid is summed feature 8 (comprising C18:1 ω7*c* and/or C18:1 ω6*c*). The major polar lipids are diphosphatidylglycerol, phosphatidylethanolamine, phosphatidylcholine, and phosphatidylglycerol. The genomic DNA G + C content of the type strain is 71.07 mol%.

The type strain IF7SW-B2^T^ is isolated from the International Space Station.

## Data Availability Statement

The 16S rRNA gene sequences of *Methylobacterium* sp. IIF1SW-B5, and *Methylobacterium* sp. IIF4SW-B5 are submitted under accession numbers KY218843 and KY218865, respectively. The WGS and the raw data deposited under BioProject accession number PRJNA634337. The WGS accession numbers are mentioned in Table 1. The WGS was also deposited in GeneLab under GeneLab dataset (GLDS-300; https://genelab-data.ndc.nasa.gov/genelab/accession/GLDS339 300). The version described in this paper is the first version.

## Author Contributions

KV and NKS conceived and designed the experiments. SB, VE, and NKS performed the experiments. NKS analyzed the genomic data inclusive of *de novo* assemblies and verification, scaffold quality assessment, and annotation and generation of the whole genome and protein level alignment for positional description of organism in the tree of life. SB independently verified the genome assembly, generated alignments for all gene trees in the manuscript, and manually curated the tree images. KV and NKS isolated the type strain, and NKS carried out the phenotypic assays and biochemical characterization. KV compiled the contribution of write-ups from all authors associated with phenotype, NKS generated genotype and tables, and SB generated phylogenetic trees and figures. VE conducted the SB generated chemotaxonomic analysis. All authors read and approved the final manuscript. CEM generated the genomic library and sequenced the genomes of all strains. CCCW and ARP reviewed the manuscript.

## Conflict of Interest

The author(s) declare that there are no conflicts of interest. This manuscript was prepared as an account of work sponsored by NASA, an agency of the US Government. The US Government, NASA, California Institute of Technology, Jet Propulsion Laboratory, and their employees make no warranty, expressed or implied, or assume any liability or responsibility for the accuracy, completeness, or usefulness of information, apparatus, product, or process disclosed in this manuscript, or represents that its use would not infringe upon privately held rights. The use of, and references to any commercial product, process, or service does not necessarily constitute or imply endorsement, recommendation, or favoring by the U.S. Government, NASA, California Institute of Technology, or Jet Propulsion Laboratory. Views and opinions presented herein by the authors of this manuscript do not necessarily reflect those of the U.S. Government, NASA, California Institute of Technology, or Jet Propulsion Laboratory, and shall not be used for advertisements or product endorsements.

## Footnotes

1 https://rast.nmpdr.org/

2 http://midi-inc.com
