# Supplementary material for: Methylobacterium ajmalii sp. nov., Isolated From the International Space Station
Source: Front Microbiol. 2021 Mar 15;12:639396. doi: 10.3389/fmicb.2021.639396 (PMC8005752; doi:10.3389/fmicb.2021.639396)

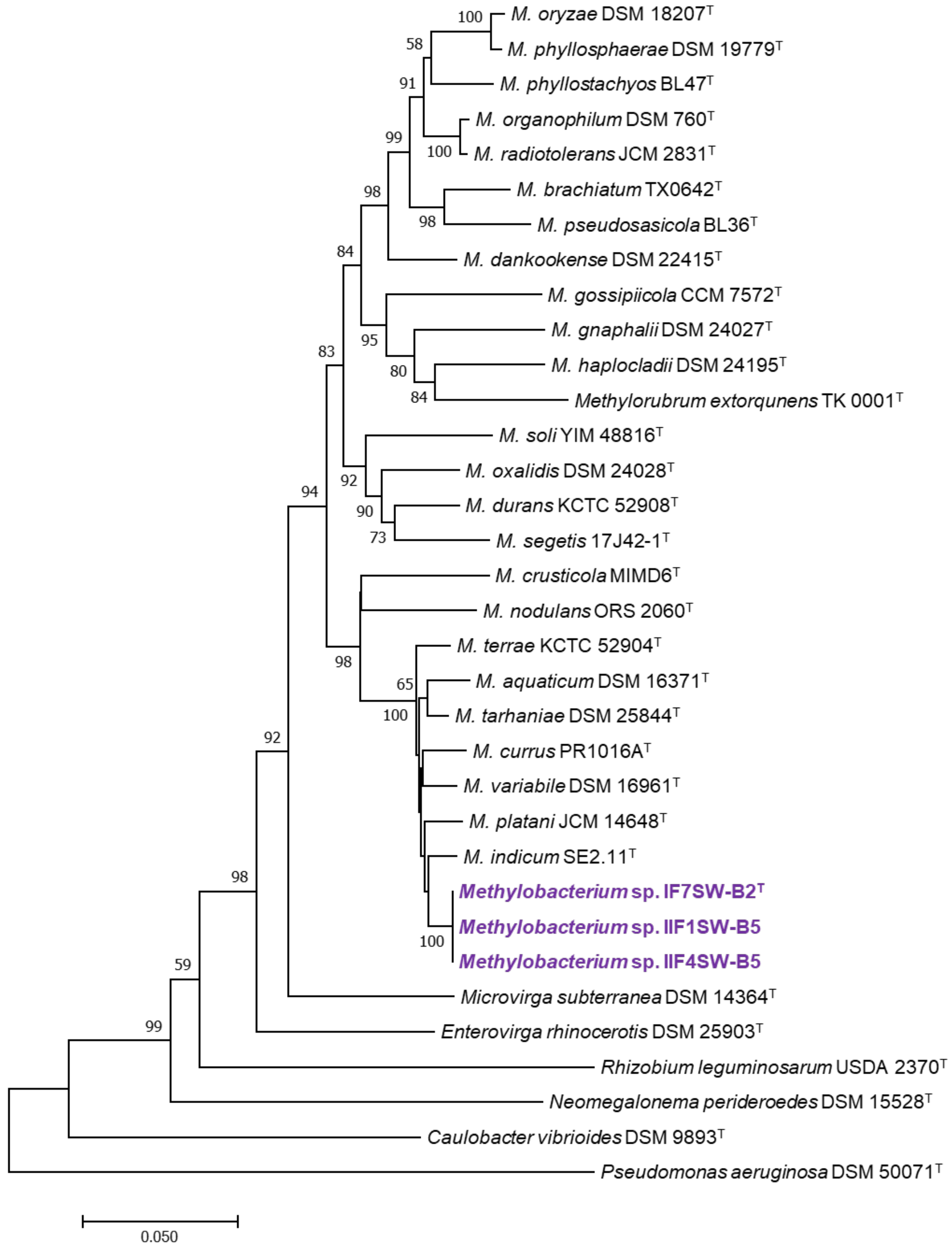

Supplementary Figure S1. Phylogenetic tree generated by the neighbor-joining method, based on ATP synthase F1 (*atpD*) beta subunit sequence, showing the phylogenetic relationship of *Methylobacterium* sp. nov. with members of the family *Methylobacteriaceae*. Bootstrap values from 1000 replications are shown at branch points. Bar, 0.05 substitution per site.

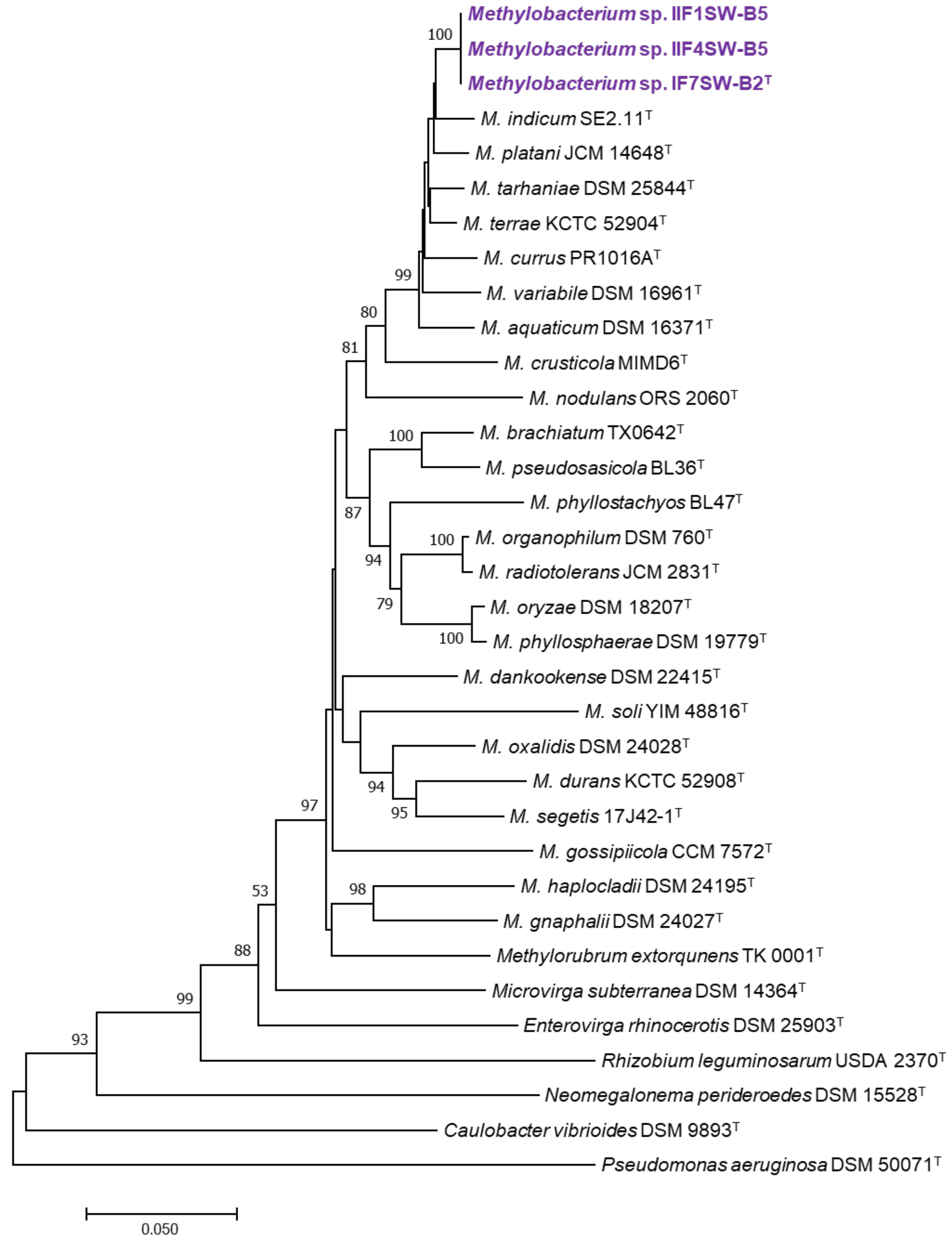

Supplementary Figure S2. Phylogenetic tree generated by the neighbor-joining method, based on DNA strand exchange and recombination gene (*recA*) sequence, showing the phylogenetic relationship of *Methylobacterium* sp. nov. with members of the family *Methylobacteriaceae*. Bootstrap values from 1000 replications are shown at branch points. Bar, 0.05 substitution per site.

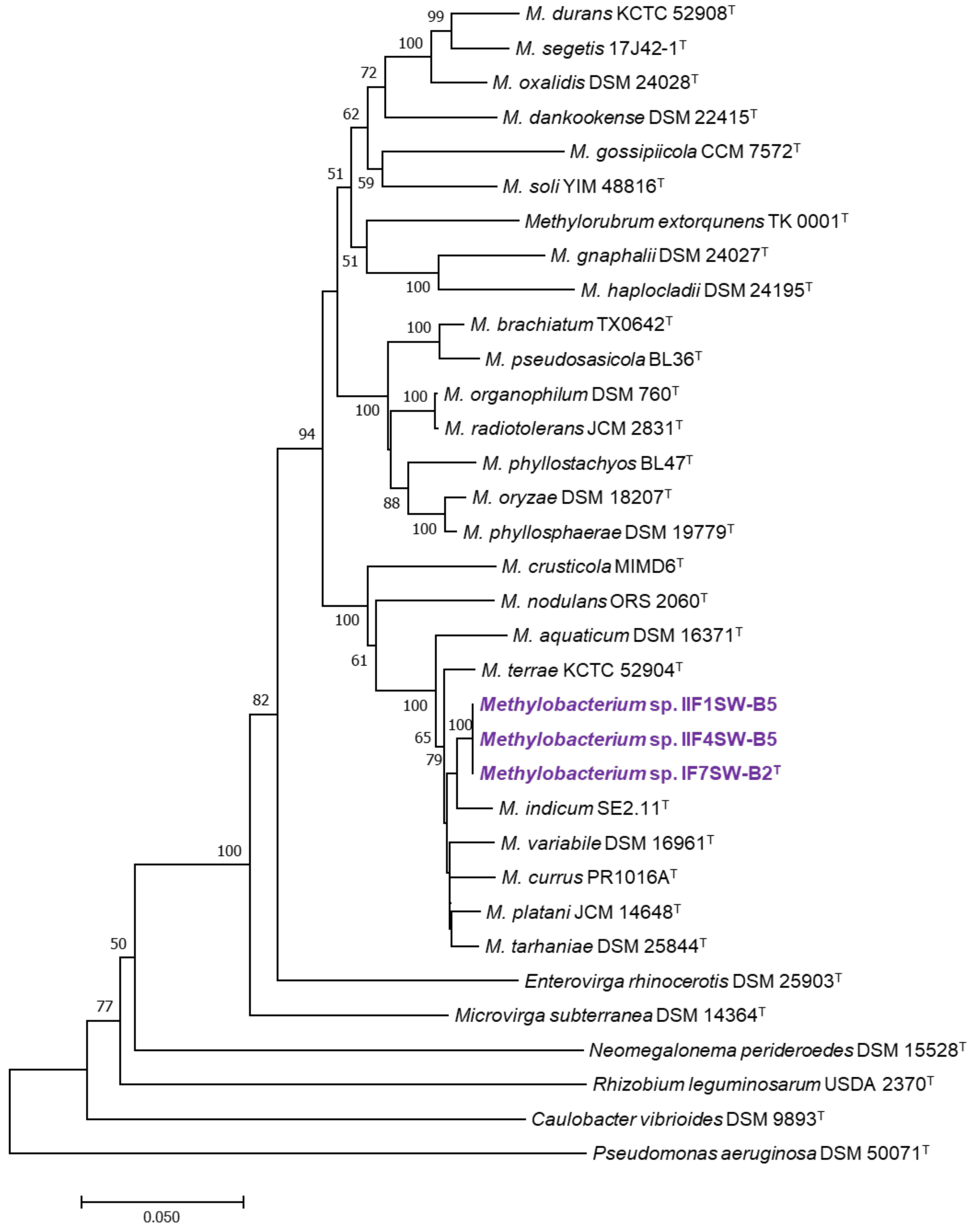

Supplementary Figure S3. Phylogenetic tree generated by the neighbor-joining method, based on chaperone gene (*dnaK*) sequence, showing the phylogenetic relationship of *Methylobacterium* sp. nov. with members of the family *Methylobacteriaceae*. Bootstrap values from 1000 replications are shown at branch points. Bar, 0.05 substitution per site.

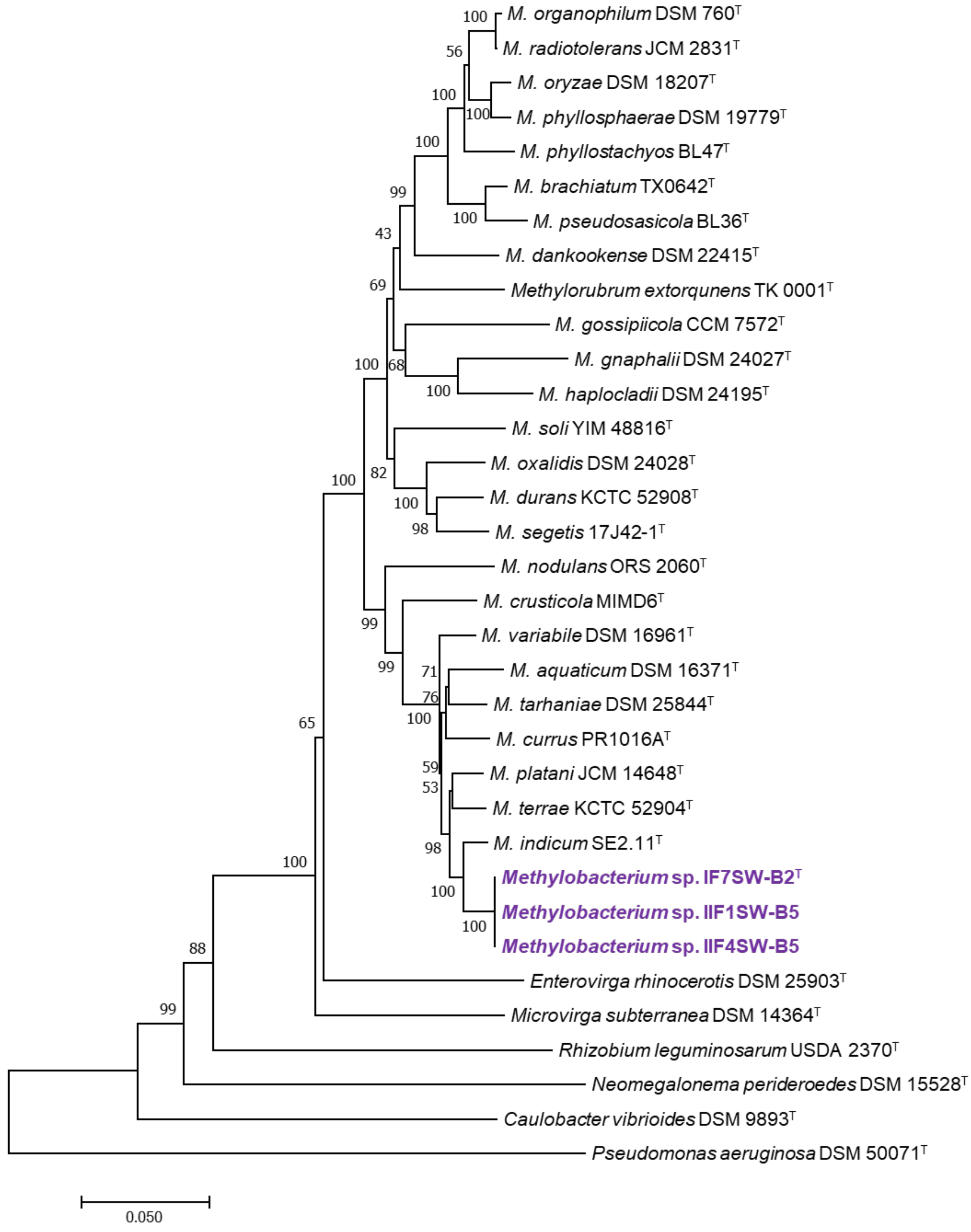

Supplementary Figure S4. Phylogenetic tree generated by the neighbor-joining method, based on DNA-directed RNA polymerase (*rpoB*) beta subunit sequence, showing the phylogenetic relationship of *Methylobacterium* sp. nov. with members of the family *Methylobacteriaceae*. Bootstrap values from 1000 replications are shown at branch points. Bar, 0.05 substitution per site.

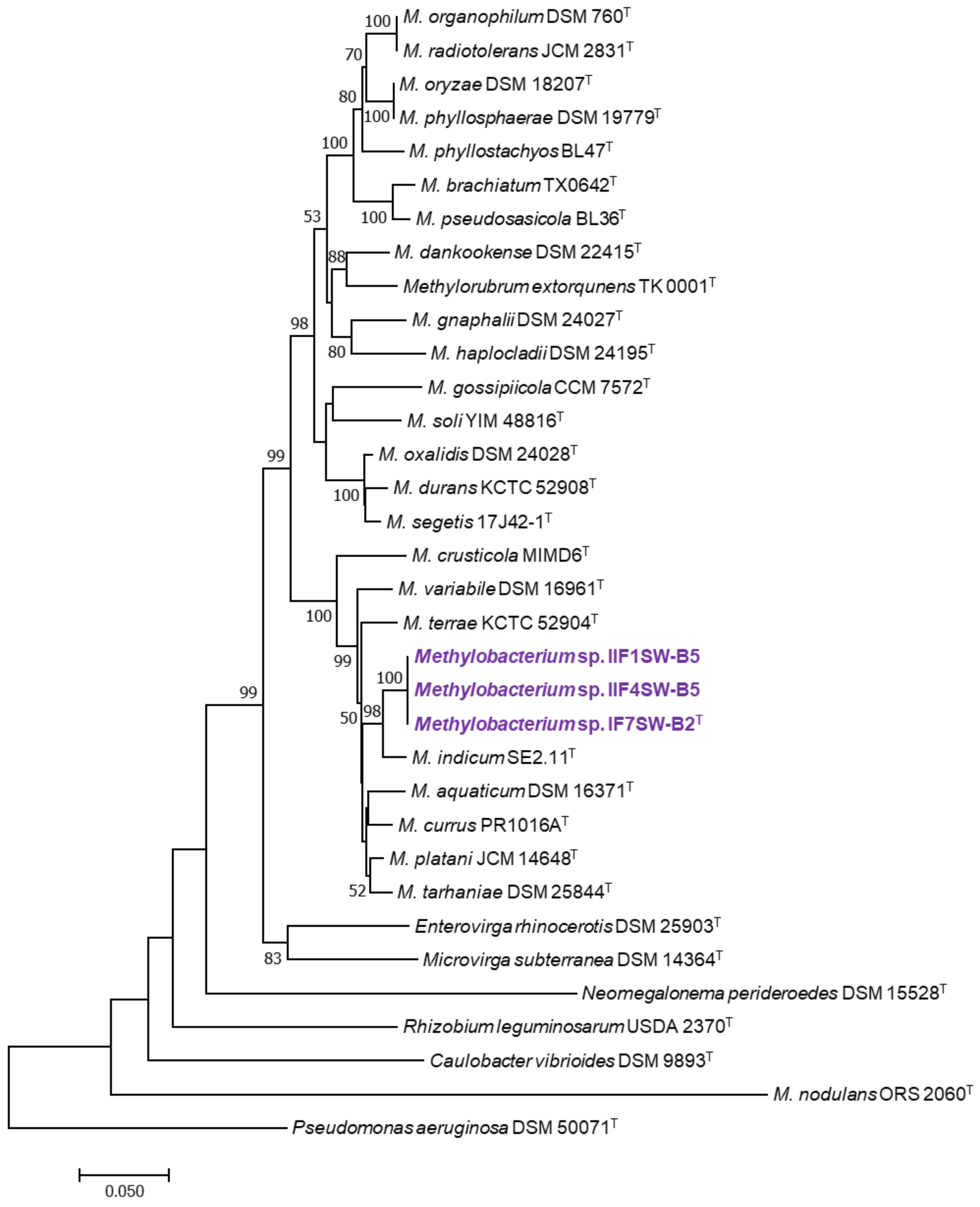

Supplementary Figure S5. Phylogenetic tree generated by the neighbor-joining method, based on glutamine synthetase type I gene (*glnI*) sequence, showing the phylogenetic relationship of *Methylobacterium* sp. nov. with members of the family *Methylobacteriaceae*. Bootstrap values from 1000 replications are shown at branch points. Bar, 0.05 substitution per site.

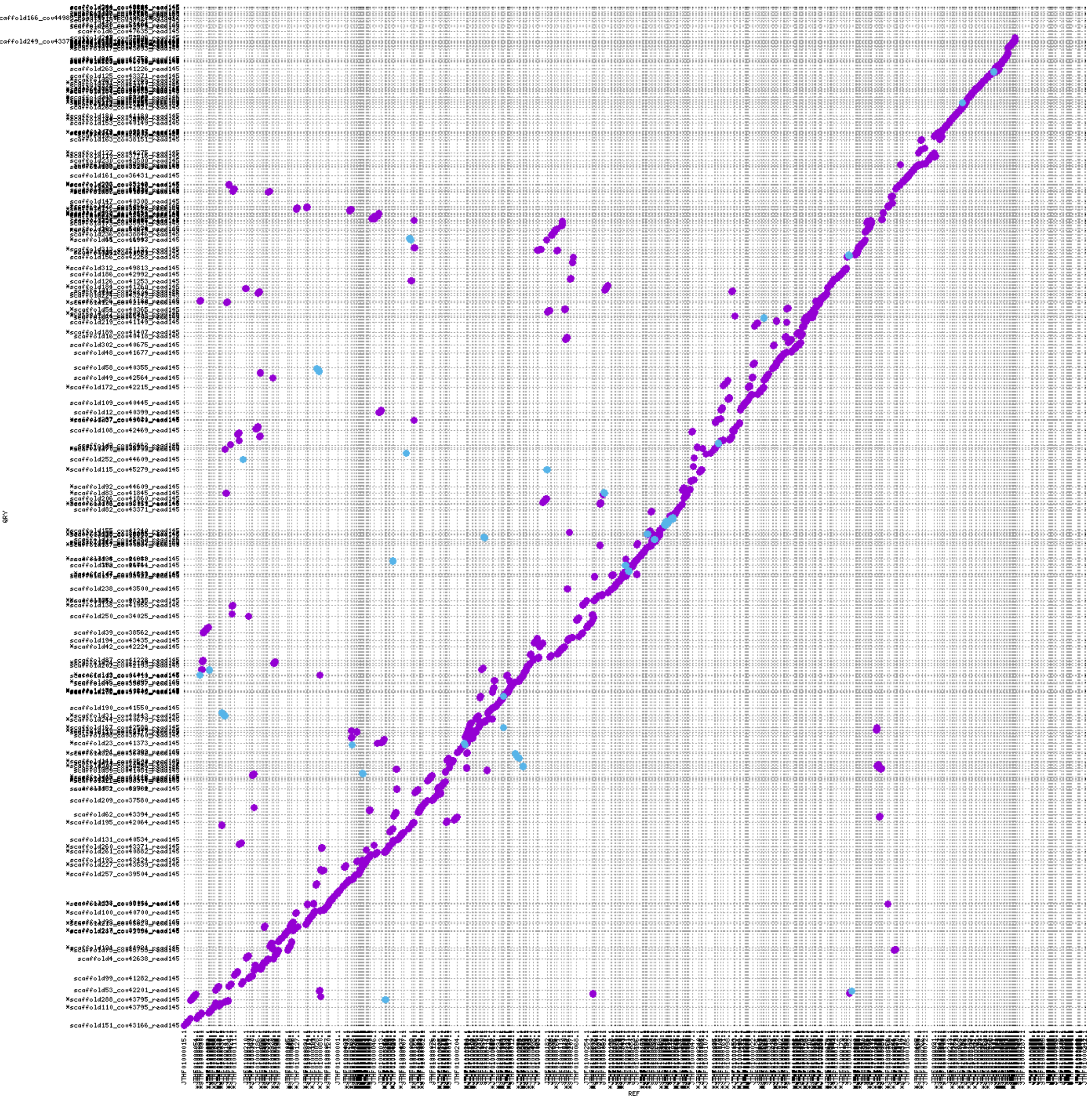

Ref: Methylobacterium_indicum_SE2.11
Query: Methylobacterium_sp._IF7SW-B2

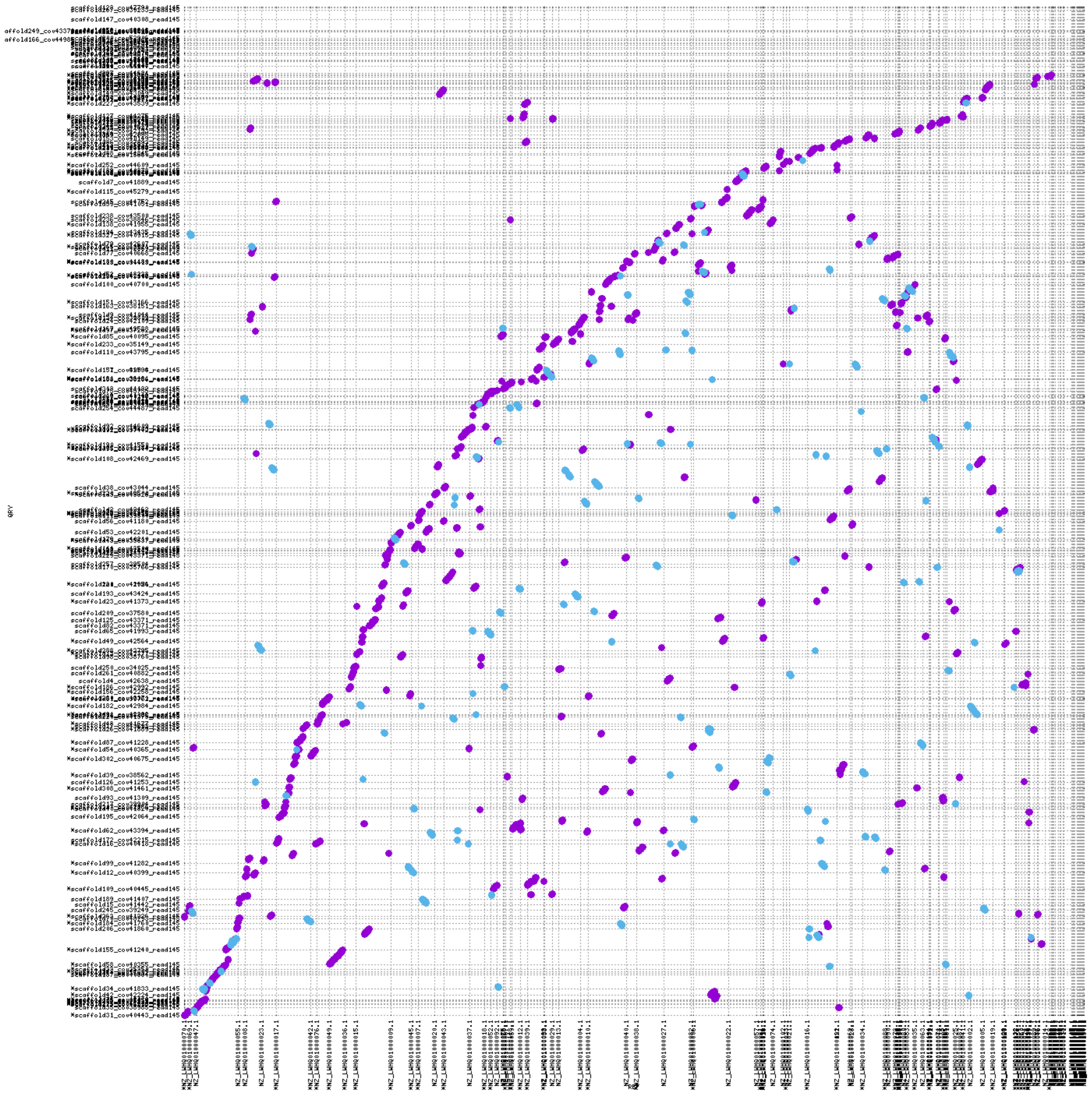

Ref: Methylobacterium_platani_PMB02
Query: Methylobacterium_sp._IF7SW-B2

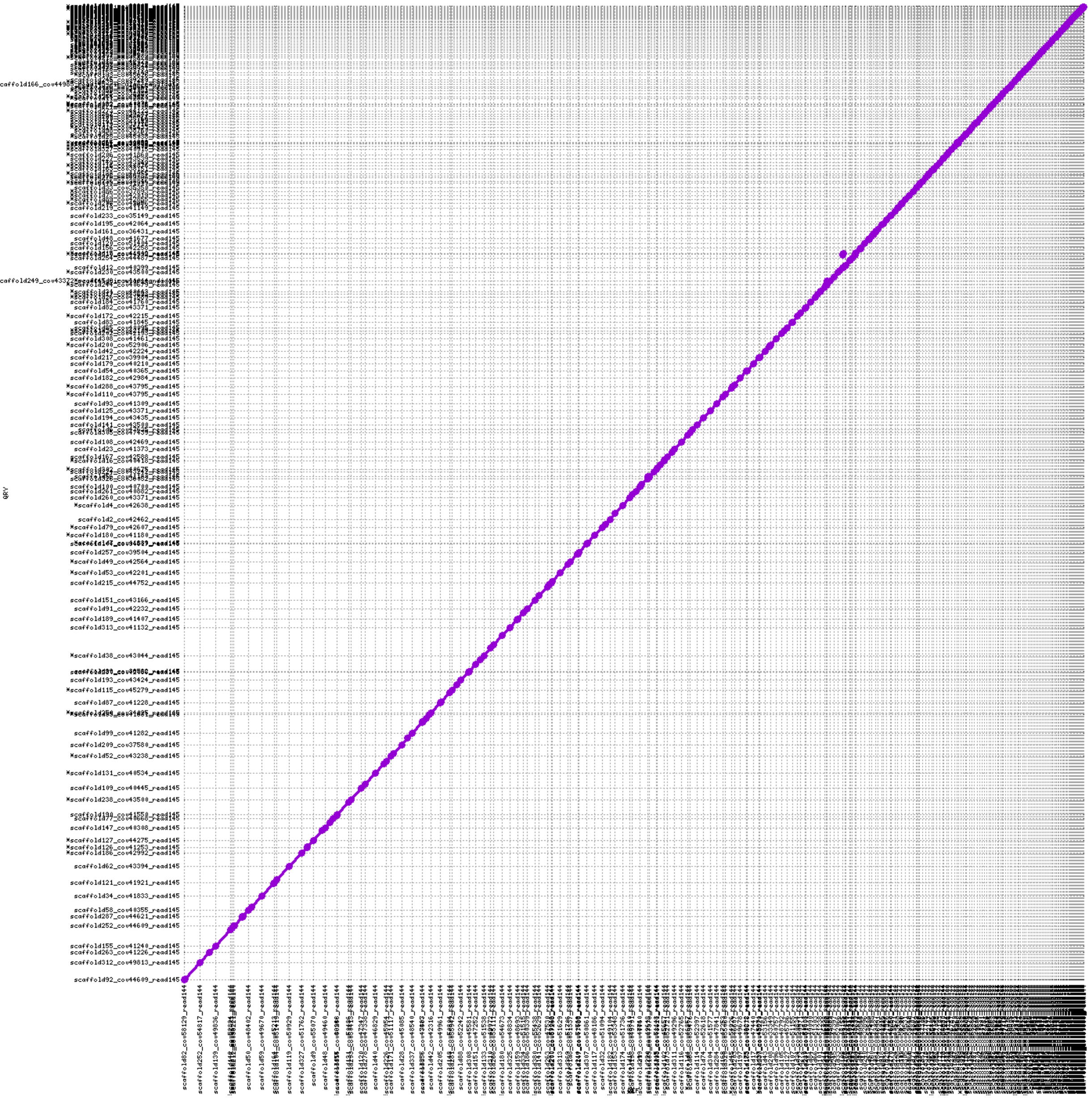

Ref: Methylobacterium_sp._IIF1SW-B5
Query: Methylobacterium_sp._IF7SW-B2

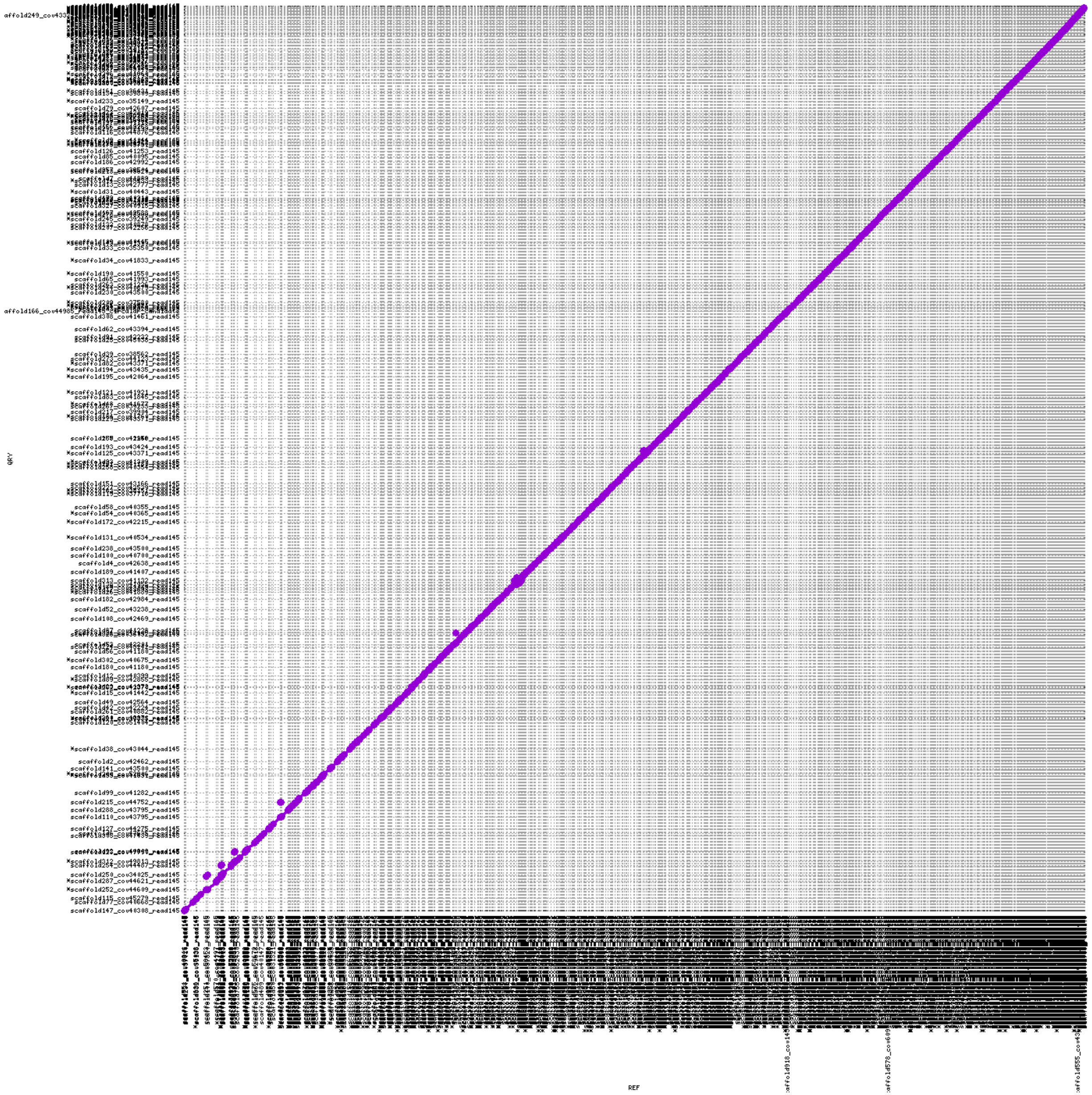

Ref: Methylobacterium_sp._IIF4SW-B5
Query: Methylobacterium_sp._IF7SW-B2

Supplementary Figure S6. MUMmer plot alignment of the entire genomes of three ISS strains, *M. indicum* SE2.11, and *M. platani* PMB02 to detect their divergence and similarity.

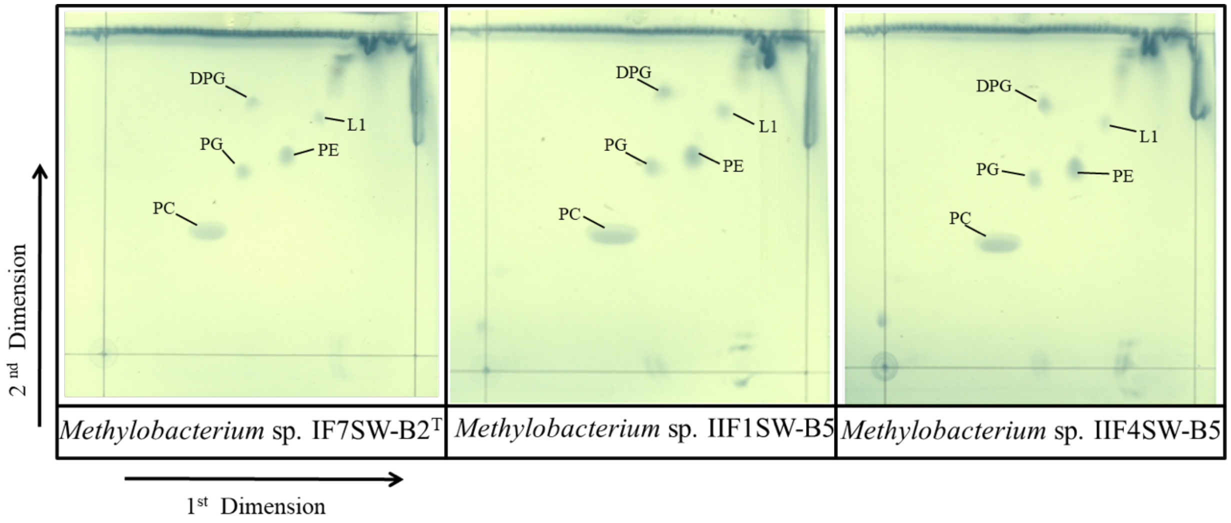

Supplementary Figure S7. Two-dimensional thin-layer chromatogram of whole cell lipid extracts of *Methylobacterium* sp. IF7SW-B2^T, *Methylobacterium* sp. IIF1SW-B5 and *Methylobacterium* sp. IIF4SW-B5. The first direction was developed in $CHCl_3$:CH_3OH:H_2O (65:25:4 v/v) and the second in $CHCl_3$:CH_3OH:CH_3COOH:H_2O (80:12:15:4 v/v). Total **polar lipids** were stained with 5% ethanolic molybdophosphoric acid. PE, phosphatidylethanolamine; DPG, diphosphatidylglycerol; PG, phosphatidylglycerol; PC, phosphatidylcholine and L1, unidentified lipid.

Supplemental Figure S8. Genes essential for interaction with plants and metabolic pathway of cobalamin predicted in the ISS IF7SW-B2^T. A thorough analysis of the IF7SW-B2^T revealed genes involved with plant interaction. miaA gene similar to *Methylobacterium extorquens* isopentenyl tRNA transferase (miaA), Multiple components of the cobalamin synthesis pathway such as Cobalamin biosynthesis protein BluB, L-threonine 3-O-phosphate decarboxylase (EC 4.1.1.81), Adenosylcobinamide-phosphate guanylyltransferase (EC 2.7.7.62), Cobyric acid synthase (EC 6.3.5.10), Nicotinate-nucleotide--dimethylbenzimidazole phosphoribosyltransferase (EC 2.4.2.21), Adenosylcobinamide-phosphate synthase (EC 6.3.1.10), Cob(I)alamin adenosyltransferase (EC 2.5.1.17), Cobalamin synthase (EC 2.7.8.26) and Adenosylcobinamide kinase (EC 2.7.1.156) were identified. Due to multiple component identification, we were able to predict the pathway for cobalamin synthesis (in red, Figure below). Genes associated with siderophore production, i.e., Ferric siderophore transport system, biopolymer transport protein ExbB, and multiple flagellar proteins were identified including FlaA, FlgA, FlgB, FlgC, FlgD, FlgE, FlgF, FlgG, FlgH, FlgI, FlgK, FlgL, FlhA, FlhB, FliD, FliE, FliF, FliG, FliH, FliI, FliJ, FliK, FliM, FliN, FliO, FliP, FliQ, FliR, FliX, FtsI, MotA, and MotB.

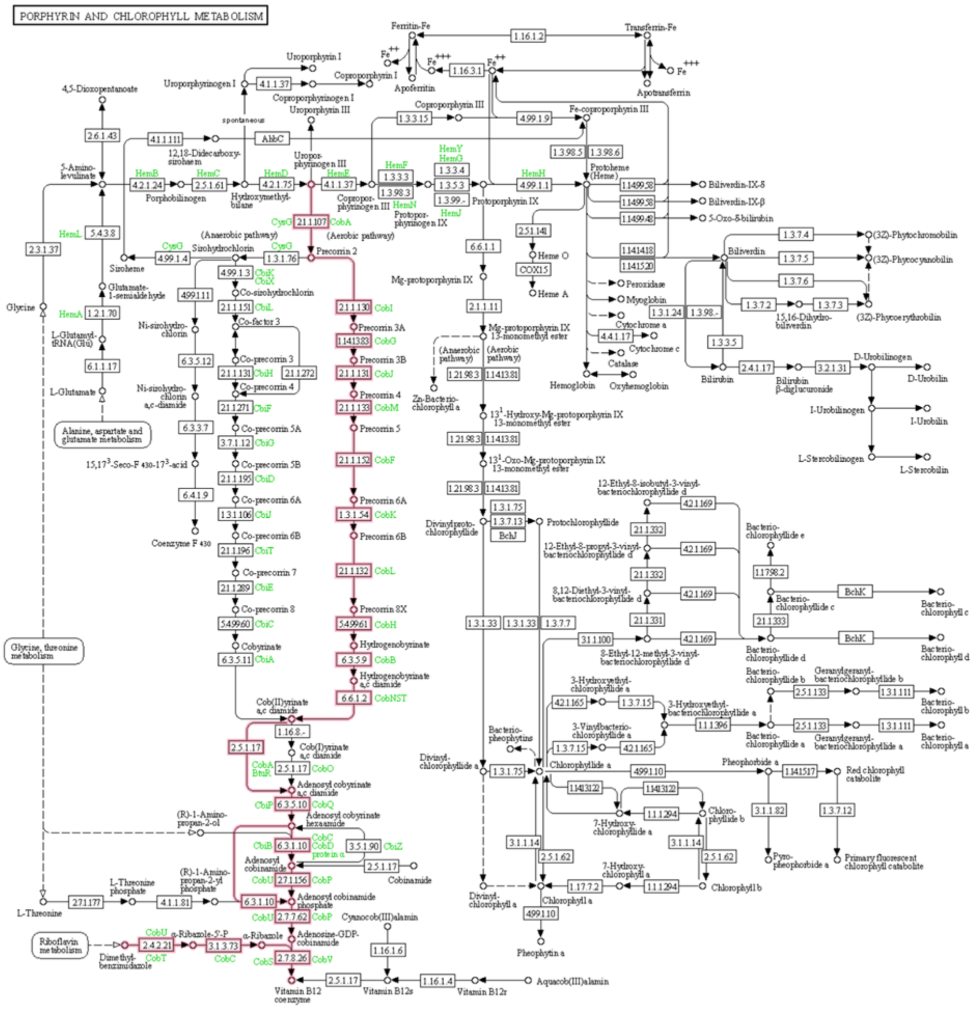

Supplement: Supplementary file 2 [file Data_Sheet_2.pdf]
