# Supplementary material for: Methylobacterium ajmalii sp. nov., Isolated From the International Space Station
Source: Front Microbiol. 2021 Mar 15;12:639396. doi: 10.3389/fmicb.2021.639396 (PMC8005752; doi:10.3389/fmicb.2021.639396)
Supplement: Supplementary file 3 [file Data_Sheet_3.pdf]

Supplemental Table S1. Pairwise comparison of 16S rRNA gene sequences of *Methylobacterium ajmalii* IF7SW-B2T and other validly described *Methylobacterium* species.

SL. No.	Name	Strain	Authors	Accession #	Pairwise Similarity(%)	Mismatch/Total nt	Completeness(%)
1	*Methylobacterium indicum*	SE2.11	Chaudhry et al. 2016	KP272100	99.34	9/1366	98.30
2	*Methylobacterium platani*	PMB02	Kang et al. 2007	LWHQ01000067	98.83	16/1367	100.00
3	*Methylobacterium aquaticum*	DSM 16371	Gallego et al. 2005	LABX01000161	98.24	24/1364	100.00
4	*Methylobacterium currus*	PR1016A	Park et al. 2018	MH158285	98.24	24/1364	100.00
5	*Methylobacterium terrae*	17Sr1-28	Kim et al. 2019	CP029553	97.95	28/1363	100.00
6	*Methylobacterium tarhaniae*	N4211	Veyisoglu et al. 2013	JQ864432	97.94	28/1359	99.72
7	*Methylobacterium variabile*	DSM 16961	Gallego et al. 2005	LABY01000145	97.80	30/1366	100.00
8	*Methylobacterium nonmethylotrophicum*	6HR-1	Feng et al. 2020	JQ608335	97.51	34/1367	100.00
9	*Methylobacterium frigidaeris*	IER25-16	Lee and Jeon 2018	KY864396	97.14	39/1362	100.00
10	*Methylobacterium terricola*	17Sr1-39	Kim et al. 2020	KY939568	96.70	45/1365	100.00
11	*Methylobacterium isbiliense*	AR24	Gallego et al. 2005	AJ888239	96.55	47/1363	98.51
12	*Methylobacterium nodulans*	ORS 2060	Jourand et al. 2004	CP001349	96.04	54/1364	100.00
13	*Methylorubrum aminovorans*	JCM 8240	(Urakami et al. 1993) Green and Ardley 2018	AB175629	95.89	56/1363	99.86
14	*Methylorubrum suomiense*	NCIMB 13778	(Doronina et al. 2002) Green and Ardley 2018	AB175645	95.81	57/1361	99.86
15	*Methylorubrum extorquens*	IAM 12631	(Urakami and Komagata 1984) Green and Ardley 2018	AB175632	95.74	58/1363	99.86
16	*Methylorubrum thiocyanatum*	DSM 11490	(Wood et al. 1999) Green and Ardley 2018	AB175646	95.73	58/1358	99.86
17	*Methylorubrum salsuginis*	MR	(Wang et al. 2007) Green and Ardley 2018	EF015478	95.66	59/1360	100.00
18	*Methylobacterium crusticola*	MIMD6	Jia et al. 2020	KT346425	95.66	59/1360	98.87
19	*Methylorubrum zatmanii*	DSM 5688	(Green et al. 1988) Green and Ardley 2018	AB175647	95.60	60/1363	99.86
20	*Methylorubrum rhodinum*	JCM 2811	(Heumann 1962) Green and Ardley 2018	BAZM01000707	95.59	60/1362	100.00
21	*Methylorubrum podarium*	FM4	(Anesti et al. 2006) Green and Ardley 2018	AF514774	95.54	60/1346	95.74
22	*Methylorubrum populi*	BJ001	(Van Aken et al. 2004) Green and Ardley 2018	CP001029	95.53	61/1365	100.00
23	*Methylobacterium durans*	17SD2-17	Kim et al. 2020	CP029550	95.45	62/1363	100.00
24	*Methylorubrum rhodesianum*	DSM 5687	(Green et al. 1988) Green and Ardley 2018	AB175642	95.45	62/1363	99.86
25	*Methylobacterium oryzihabitans*	TER-1	Chen et al. 2019	MF980971	95.38	63/1363	100.00
26	*Methylorubrum pseudosasae*	BL44	(Madhaiyan and Poonguzhali 2016) Green and Ardley 2018	EU912442	95.13	66/1355	97.80
27	*Methylobacterium planium*	YIM 132548	Jiang et al. 2020	MN317338	94.95	69/1366	100.00
28	*Methylobacterium oxalidis*	35a	Tani et al. 2012	AB607860	94.87	70/1365	100.00
29	*Methylobacterium soli*	YIM 48816	Cao et al. 2013	EU860984	94.87	70/1364	99.29
30	*Methylobacterium iners*	5317S-33	Weon et al. 2008	EF174497	94.84	69/1336	96.37
31	*Methylobacterium pseudosasicola*	BL36	Madhaiyan and Poonguzhali 2014	jgi.1071178	94.80	71/1365	100.00
32	*Methylobacterium segetis*	17J42-1	Ten et al. 2019	LC433921	94.80	71/1365	100.00
33	*Methylobacterium adhaesivum*	AR27	Gallego et al. 2006	AM040156	94.80	71/1365	98.58
34	*Methylobacterium gossipiicola*	Gh-105	Madhaiyan et al. 2012	jgi.1071170	94.79	71/1363	100.00
35	*Methylobacterium organophilum*	ATCC 27886	Patt et al. 1976	AB175638	94.71	72/1361	99.86
36	*Methylobacterium mesophilicum*	JCM 2829	(Austin and Goodfellow 1979) Green and Bousfield 1983	D32225	94.64	73/1363	100.00
37	*Methylobacterium marchantiae*	JT1	Schauer et al. 2011	FJ157976	94.61	73/1354	99.36
38	*Methylobacterium bullatum*	F3.2	Hoppe et al. 2012	GU983169	94.58	73/1348	98.79
39	*Methylobacterium goesingense*	iEII3	Idris et al. 2012	AY364020	94.57	74/1363	100.00
40	*Methylobacterium brachiatum*	B0021	Kato et al. 2008	AB175649	94.56	74/1361	99.86
41	*Methylobacterium persicinum*	002-165	Kato et al. 2008	AB252202	94.50	75/1363	99.86
42	*Methylobacterium komagatae*	002-079	Kato et al. 2008	AB252201	94.21	79/1365	99.86
43	*Methylobacterium hispanicum*	GP34	Gallego et al. 2005	AJ635304	94.13	80/1362	99.57
44	*Methylobacterium* sp.	IF7SW-B2T	This study		100.00	0/1444	100.00
45	*Methylobacterium* sp.	IIF1SW-B5	This study		100.00	0/1444	100.00
46	*Methylobacterium* sp.	IIF4SW-B5	This study		100.00	0/1444	100.00

Supplemental Table S2. Classification and general features of *Methylobacterium ajmalii* strain IF7SW-B2^{T} according to the MIGS recommendations [1].

MIGS ID	Property	Term	Evidence code[a]
	Current classification	Domain *Bacteria*	TAS [2]
		Phylum *Proteobacteria*	TAS [3]
		Class *Alphaproteobacteria*	TAS [4]
		Order *Rhizobiales*	TAS [5]
		Family *Methylobacteriaceae*	TAS [6]
		Genus *Methylobacterium*	IDA
		Species *ajmalii*	IDA
		Type strain IF7SW-B2^{T}	IDA
	Gram stain	Negative	IDA
	Cell shape	Rod-shaped	IDA
	Motility	Motile	IDA
	Sporulation	Nonsporulating	IDA
	Temperature range	Mesophilic	IDA
	Optimum	25˚C to 30˚C	IDA
MIGS-6.3	Salinity	1% NaCl	IDA
MIGS-22	Oxygen requirement	Aerobic	IDA
	Carbon source	Unknown	NAS
	Energy source	Unknown	NAS
MIGS-6	Habitat	ISS	IDA
MIGS-15	Biotic relationship	Free-living	IDA
	Pathogenicity	Unknown	
	Biosafety level	1	
MIGS-14	Isolation	Lab 3 overhead	IDA
MIGS-4	Geographic location	Pasadena, CA, USA	IDA
MIGS-5	Sample collection time	March 04, 2015	IDA
MIGS-4.1	Latitude	34.1478° N	IDA
MIGS-4.2	Longitude	118.1445° W	IDA
MIGS-4.3	Depth	0 m	IDA
MIGS-4.4	Altitude	272 meter above sea level	IDA

[a]Evidence codes - IDA: Inferred from Direct Assay; TAS: Traceable Author Statement (i.e., a direct report exists in the literature); NAS: Non-traceable Author Statement (i.e., not directly observed for the living, isolated sample but based on a generally accepted property for the species or anecdotal evidence). Evidence codes come from the Gene Ontology project. If the evidence is IDA, then the property was directly observed for a live isolate by one of the authors or an expert mentioned in the acknowledgements.

1. Field D, Garrity G, Gray T, Morrison N, Selengut J, Sterk P, Tatusova T, Thomson N, Allen MJ, Angiuoli SV *et al*: **The minimum information about a genome sequence (MIGS) specification**. *Nat Biotechnol* 2008, **26**(5):541-547.
2. Woese CR, Kandler O, Wheelis ML: **Towards a natural system of organisms: proposal for the do mains Archaea, Bacteria, and Eucarya**. *Proc Natl Acad Sci USA* 1990, **87**.
3. **Proteobacteria phyl. nov**. In: *Bergey's Manual of Systematics of Archaea and Bacteria.* 1-1.
4. **Alphaproteobacteria class. nov**. In: *Bergey's Manual of Systematics of Archaea and Bacteria.* 1-1.
5. **Rhizobiales ord. nov**. In: *Bergey's Manual of Systematics of Archaea and Bacteria.* 1-1.
6. **Methylobacteriaceae fam. nov**. In: *Bergey's Manual of Systematics of Archaea and Bacteria.* 1-1.

Supplementary Table S3. Phenotypic analysis of *Methylobacterium* nov. sp. using API 20NE strip.

Strains: 1: *Methylobacterium* sp. IF7SW-B2 ^{T} (this study), 2: *Methylobacterium* sp. IIF1SW-B5 (this study); 3: *Methylobacterium* sp. IIF4SW-B5 (this study). w: weakly positive; +: Positive; -: Negative.

**Feature**	**1**	**2**	**3**
Nitrate reduction	-	-	-
Indole production	-	-	-
D-glucose fermentation	-	-	-
L-arginine Dihydrolase	-	-	-
Urease	+	+	+
Esculine/ ferric citrate hydrolysis (β-glucosidase)	w	w	w
Gelatin hydrolysis (protease)	-	-	-
4-nitrophenyl- βD-galactopyranoside (β-galactosidase)	-	-	-
D-glucose assimilation	+	+	+
L-arabinose assimilation	+	+	+
D-mannose assimilation	+	+	+
D-mannitol assimilation	+	+	+
N-acetyl-glucosamine assimilation	+	+	+
D-maltose assimilation	+	+	+
Potassium gluconate assimilation	+	+	+
Capric acid assimilation	-	-	-
Adipic acid assimilation	+	+	+
Malic acid assimilation	+	+	+
Trisodium citrate assimilation	+	+	+
Phenyl acetic acid assimilation	-	-	-
Cytochrome oxidase	+	+	+

Supplementary Table S4. Phenotypic analysis of *Methylobacterium ajmalii* nov. sp. using API ZYM strip.

Strains: 1: *Methylobacterium* sp. IF7SW-B2^{T} (this study), 2: *Methylobacterium* sp. IIF1SW-B5 (this study); 3: *Methylobacterium* sp. IIF4SW-B5 (this study). w: weakly positive; +: Positive; -: Negative.

**Feature**	**1**	**2**	**3**
Alkaline phosphatase	+	+	+
Esterase (C 4)	+	+	+
Esterase lipase (C 8)	+	+	+
Lipase (C 14)	-	-	-
Leucine arylamidase	+	+	+
Valine arylamidase	-	-	-
Cystine arylamidase	-	-	-
Trypsin	+	+	+
α-chymotrypsin	-	-	-
Acid phosphatase	+	+	+
Naphtol-AS-BI-phosphohydrolase	+	+	+
α-galactosidase	-	-	-
β-galactosidase	-	-	-
β-glucuronidase	-	-	-
β-glucosidase	-	-	-
α-glucosidase	-	-	-
N-acetyl-β-glucosaminidase	-	-	-
α-mannosidase	-	-	-
α-fucosidase	-	-	-

Supplementary Table S5. Phenotypic analysis of *Methylobacterium ajmalii* nov. sp. using API 50 CH strip.

Strains: 1: *Methylobacterium* sp. IF7SW-B2 ^{T} (this study), 2: *Methylobacterium* sp. IIF1SW-B5 (this study); 3: *Methylobacterium* sp. IIF4SW-B5 (this study). w: weakly positive; +: Positive; -: Negative.

**Feature**	**1**	**2**	**3**
Glycerol	-	-	-
Erythritol	-	-	-
D-arabinose	-	-	-
L-arabinose	-	-	-
D-ribose	-	-	-
D-xylose	-	-	-
L-xylose	-	-	-
ADO	-	-	-
Methyl-beta-D-xylopyranoside	-	-	-
D-galactose	-	-	-
D-glucose	-	-	-
D-fructose	-	-	-
D-mannose	-	-	-
L-sorbose	-	-	-
L-rhamnose	-	-	-
Dulcitol	-	-	-
Inositol	-	-	-
D-mannitol	-	-	-
D-sorbitol	-	-	-
Methyl-alpha-D-mannopyranoside	-	-	-
Methyl-alpha-D-glucopyranoside	-	-	-
N-acetylglucosamine	-	-	-
Amygdalin	-	-	-
Arbutin	-	-	-
Esculin ferric citrate	-	-	-
Salicin	-	-	-
D-cellobiose	-	-	-
D-maltose	-	-	-
D-lactose (bovine origin)	-	-	-
D-mellibiose	-	-	-
D-saccharose (sucrose)	-	-	-
D-trehalose	-	-	-
Inulin	w	w	w
D-melezitose	w	w	w
D-raffinose	-	-	-
Amidon (starch)	-	-	-
Glycogen	-	-	-
Xylitol	-	-	-
Gentiobiose	-	-	-
D-turanose	-	-	-
D-lyxose	-	-	-

D-tagatose	-	-	-
D-fucose	-	-	-
L-fucose	-	-	-
D-arabitol	-	-	-
L-arabitol	-	-	-
Potassium gluconate	-	-	-
Potassium 2-ketogluconate	-	-	-
Potassium 5-ketogluconate	-	-	-
